# Integrated Phytochemical and Pharmacological Insights into *Premna serratifolia* for Drug Discovery: A Narrative Review

**DOI:** 10.3390/plants15142176

**Published:** 2026-07-15

**Authors:** Alfred Ariyanto, Wisnu Ananta Kusuma, Muhaimin Muhaimin, Ezatul Ezleen Kamarulzaman, Taufik Muhammad Fakih, Muchtaridi Muchtaridi

**Affiliations:** 1 Doctoral Program of Pharmacy, Faculty of Pharmacy, Universitas Padjadjaran, Jalan Raya Bandung-Sumedang KM 21, Sumedang 45363, Indonesia; alfred23001@mail.unpad.ac.id; 2 Department of Pharmaceutical Analysis and Medicinal Chemistry, Faculty of Pharmacy, Universitas Padjadjaran, Jalan Raya Bandung-Smeeding KM 21, Sumedang 45363, Indonesia; taufikmuhammadf@unisba.ac.id; 3 School of Data Science, Mathematics, and Informatics, IPB University, Jalan Raya Dramaga, Bogor 16680, Indonesia; ananta@apps.ipb.ac.id; 4 Department of Biological Pharmacy, Faculty of Pharmacy, Universitas Padjadjaran, Jalan Raya Bandung-Sumedang KM 21, Jatinangor 45363, Indonesia; muhaimin@unpad.ac.id; 5 School of Pharmaceutical Sciences, Universiti Sains Malaysia, Jalan Sungai Dua, Minden 11800, Penang, Malaysia; ezatulezleen@usm.my; 6 Department of Pharmacy, Faculty of Mathematics and Natural Sciences, Universitas Islam Bandung, Jalan Ranggagading, Bandung 40116, Indonesia; 7 Research Collaboration Centre for Radiopharmaceuticals Theranostics, National Research and Innovation Agency (BRIN), Jalan Raya Bandung-Sumedang KM 21, Sumedang 45363, Indonesia

**Keywords:** *Premna serratifolia* Linn, bioactive compounds, network pharmacology, drug-likeness, natural product development

## Abstract

Natural products remain a rich source of chemically diverse molecules with therapeutic potential, and *Premna serratifolia* represents one such promising botanical resource. This review provides a comprehensive overview of its bioactive constituents by integrating published evidence related to extraction variability, chemical profiling, physicochemical screening, and pharmacological activities. Available studies indicate that differences in extraction techniques influence the distribution of secondary metabolites, particularly phenolic and flavonoid compounds associated with biological activity. Previous phytochemical investigations have reported a structurally diverse set of compounds spanning multiple chemical classes. Evaluations based on drug-relevant parameters suggest that several constituents possess favorable structural and physicochemical characteristics. Published studies further suggest that selected compounds may interact with multiple protein targets involved in disease-related pathways. Computational evidence reported in the literature highlights potential molecular interactions and signaling networks associated with therapeutic activities. These pathways have been linked to biological processes such as cellular regulation, oxidative balance, and inflammatory responses. Available experimental evidence indicates that several compounds exhibit noteworthy bioactivities across different biological systems. Structural features, including functional group distribution and degree of substitution, have been reported to influence molecular interactions and pharmacokinetic properties. Simpler molecular frameworks often appear to show greater compatibility with drug-likeness criteria. The integration of phytochemical, pharmacological, and computational evidence provides a useful framework for compound prioritization and evaluation. Such an approach may facilitate the transition from phytochemical discovery toward pharmacological development. Further studies involving structural optimization, advanced computational modeling, and experimental validation are warranted to better characterize the therapeutic potential of these compounds. The available evidence suggests that *Premna serratifolia* is a valuable source of bioactive molecular scaffolds with considerable potential for future multitarget drug discovery and therapeutic development.

## 1. Introduction

**Figure 1:**
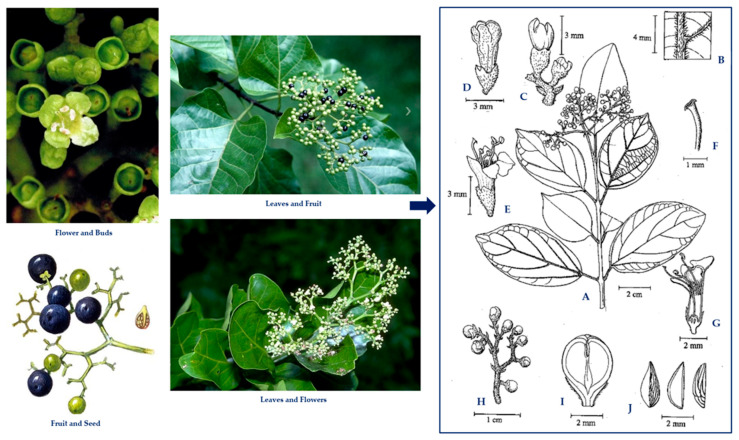
Morphological, Macroscopic, and Anatomical Characteristics of *Premna serratifolia*. (A) Flowering branch; (B) stem trichomes; (C) flower bud; (D) floral bud; (E) open flower; (F) pistil; (G) longitudinal section of flower; (H) infructescence; (I) longitudinal section of fruit; (J) seed [3,24].

*Premna serratifolia* is a medicinal plant that has gained considerable attention in recent years due to its remarkable phytochemical diversity and promising pharmacological potential across multiple therapeutic areas. This species is widely distributed in tropical and subtropical regions, where it grows in coastal, lowland, and forest-edge environments that support diverse ecological adaptations [1,2]. Figure 1 presents the morphological, macroscopic, and anatomical characteristics of *Premna serratifolia*, including its clustered small flowers, serrated leaves, fruit structures, seeds, and internal anatomical features, which are essential for accurate botanical identification and pharmacognostic validation [3]. The plant typically exhibits opposite leaves with serrated margins, small tubular flowers arranged in clusters, and drupe-like fruits containing seeds that contribute to its reproductive identification. Accurate identification is a fundamental requirement to ensure consistency in phytochemical and pharmacological investigations [4,5]. Phytochemical studies have revealed that *Premna serratifolia* contains a wide range of secondary metabolites, including flavonoids, iridoid glycosides, phenylpropanoid glycosides, terpenoids, and simple phenolic compounds [6]. These compounds exhibit diverse chemical scaffolds characterized by aromatic rings, hydroxyl groups, and glycosidic linkages. Such structural diversity enables interaction with multiple biological targets, which is advantageous for treating multifactorial diseases [7]. Functional groups such as hydroxyl and methoxy moieties influence binding affinity, reactivity, and solubility. Aromatic systems further enhance ligand–protein interactions through π–π stacking and hydrophobic contacts [8,9]. Environmental factors such as soil composition, climate, and altitude significantly affect metabolite accumulation within the plant. Variations in plant parts, including leaves, stems, and fruits, also contribute to differences in phytochemical composition [10]. These variations lead to differences in biological activity observed across studies. While this diversity enhances therapeutic potential, it also introduces challenges in identifying key active compounds [11]. The complexity of its chemical profile requires systematic evaluation to understand structure–activity relationships [12]. A comprehensive approach integrating botanical, chemical, and pharmacological data is therefore necessary. This integration supports the identification of promising bioactive compounds with therapeutic relevance.

The traditional use of *Premna serratifolia* in ethnomedicine reflects its long-standing role in treating inflammatory conditions, infections, and metabolic disorders. Modern pharmacological studies have confirmed several of these traditional uses by demonstrating antioxidant, anti-inflammatory, antimicrobial, and anticancer activities associated with its extracts and isolated compounds [13,14]. These biological activities are largely influenced by phenolic and flavonoid constituents that regulate oxidative stress and inflammatory pathways [15]. Experimental findings have shown that these compounds can modulate key cellular processes such as apoptosis, immune response, and cell proliferation [16]. Additional studies have reported neuroprotective, hepatoprotective, and cardioprotective effects in experimental models [17]. The wide range of biological activities highlights the multitarget nature of the plant’s phytochemical constituents. Different extraction techniques, including maceration, Soxhlet extraction, and decoction, have been used to isolate bioactive compounds with varying efficiency. The choice of solvent polarity significantly affects the extraction of specific compound classes, with polar solvents favoring phenolics and flavonoids [18,19]. Extraction temperature and duration also influence compound stability and yield [20]. Analytical techniques have enabled the comprehensive identification and characterization of metabolites. These methods provide both qualitative and quantitative insights into the chemical composition of plant extracts [21]. Advanced profiling techniques allow the detection of minor compounds that may contribute significantly to biological activity. Computational approaches have further enhanced the understanding of compound–target interactions. These methods facilitate the identification of key signaling pathways associated with disease mechanisms [22,23]. The integration of computational predictions with experimental validation strengthens the interpretation of pharmacological effects. Despite these advances, challenges remain in selecting compounds with optimal drug-like properties. Therefore, further studies are required to prioritize the most promising candidates for development.

Mapping the metabolite profile of bioactive compounds is a critical step in predicting pharmacological activity and guiding drug discovery efforts. Comprehensive profiling enables the identification of compounds that contribute significantly to observed biological effects [25]. Structural features such as molecular weight, polarity, hydrogen-bonding capacity, and substitution patterns influence pharmacokinetic behavior and target interaction. Drug-likeness evaluation based on Lipinski’s rule of five, lead-likeness, lipophilicity, and synthetic accessibility helps identify compounds suitable for development [26]. Compounds that meet these criteria are more likely to exhibit favorable absorption and bioavailability. Computational approaches provide insights into molecular interactions and binding stability. These methods allow identification of key targets and pathways involved in disease modulation [27,28]. Pathway analysis highlights involvement in processes such as inflammation, oxidative stress, and cell proliferation. The integration of metabolite profiling with computational modeling improves the accuracy of pharmacological prediction. This combined approach supports rational selection of compounds for further investigation. In vitro studies provide validation of predicted biological activities at the cellular level [29]. In vivo studies further confirm therapeutic efficacy, pharmacokinetics, and safety [30]. The combination of analytical, computational, and experimental strategies enhances the efficiency of natural product-based drug discovery. Such an integrated framework allows systematic evaluation from plant extraction to biological validation. This approach is particularly important for complex plants with diverse metabolite profiles. The ability to accurately map and interpret these profiles will determine the success of future drug development efforts. These strategies support the advancement of *Premna serratifolia* as a valuable source of bioactive compounds with therapeutic potential.

Despite the growing number of studies investigating the phytochemical composition and biological activities of *Premna serratifolia*, the available information remains fragmented across botanical, phytochemical, pharmacological, and computational research domains. Previous reports have primarily focused on ethnomedicinal applications, extract-based biological activities, or specific classes of secondary metabolites, with limited efforts to integrate phytochemical diversity, metabolite profiling, drug-likeness assessment, ADMET characteristics, structural feature analysis, and target-oriented pharmacological evidence within a unified framework.

Furthermore, no previous narrative review has comprehensively evaluated the relationships between identified metabolites, their physicochemical properties, computational predictions, and experimentally reported pharmacological activities. Therefore, this review aims to provide an integrated overview of the phytochemical diversity, biological activities, structural characteristics, drug-likeness properties, ADMET profiles, and pharmacological evidence associated with *Premna serratifolia*. In addition, the review highlights current limitations, clearly distinguishes direct plant-based evidence from indirect compound-level evidence, and discusses future directions for the development of *Premna serratifolia*-derived bioactive compounds.

## 2. Review Methodology

This narrative review was conducted to provide a comprehensive summary and critical evaluation of current knowledge regarding the phytochemical composition, pharmacological activities, molecular targets, and drug discovery potential of *Premna serratifolia*. A structured literature search was performed using major scientific databases, including PubMed, Scopus, Google Scholar, ScienceDirect, SpringerLink, Wiley Online Library, Taylor & Francis Online, SAGE Journals, MDPI, and Dovepress. The search encompassed articles published up to May 2026, with particular emphasis placed on studies published over the last two decades to capture both foundational and recent advances in phytochemistry, pharmacology, and computational drug discovery. The search strategy employed combinations of keywords and Boolean operators, including “*Premna serratifolia*”, “phytochemistry”, “secondary metabolites”, “bioactive compounds”, “pharmacological activity”, “anti-inflammatory”, “antioxidant”, “antimicrobial”, “anticancer”, “molecular target”, “network pharmacology”, “ADMET”, “drug discovery”, and related terms. The inclusion criteria comprised peer-reviewed original research articles, review articles, ethnopharmacological studies, phytochemical investigations, pharmacological evaluations, computational studies, and relevant methodological reports published in English. Studies were included if they reported information on the chemical constituents of *Premna serratifolia*, its biological activities, molecular mechanisms, target proteins, pharmacokinetic properties, toxicity profiles, or therapeutic applications.

The initial literature search yielded approximately 3919 records, consisting of 17 records from PubMed, 85 from Scopus, 2700 from Google Scholar, 107 from ScienceDirect, 125 from SpringerLink, 58 from Wiley Online Library, 820 from Taylor & Francis Online, 4 from SAGE Journals, 2 from MDPI, and 1 from Dovepress. After removal of duplicate records and preliminary screening based on titles and abstracts, studies unrelated to *Premna serratifolia*, inaccessible conference abstracts, and articles lacking sufficient methodological information were excluded. Additional relevant references were identified through manual screening of the reference lists of eligible articles to ensure comprehensive coverage of the available evidence. Following full-text assessment according to the predefined inclusion and exclusion criteria, eligible studies were selected and incorporated into the final narrative synthesis.

Data extraction and organization were performed systematically based on predefined thematic categories. Information relating to phytochemical constituents, chemical classifications, plant parts investigated, extraction methods, pharmacological activities, molecular targets, mechanisms of action, ADMET characteristics, and therapeutic indications was collected, categorized, and synthesized. Particular attention was given to studies reporting compounds with multitarget pharmacological effects and those demonstrating potential relevance for contemporary drug discovery approaches. The extracted evidence was critically evaluated and integrated to identify major bioactive constituents, key pharmacological pathways, emerging therapeutic opportunities, existing research gaps, and future directions for the development of *Premna serratifolia*-derived drug candidates. Although this study was conducted as a narrative review rather than a systematic review, a structured literature selection approach was adopted to enhance the transparency and reproducibility of the evidence synthesis. This approach provides a comprehensive and transparent framework for understanding the potential of *Premna serratifolia* as a promising source of multitarget therapeutic agents.

## 3. Biological Activities of *Premna serratifolia*

The biological activities of *Premna serratifolia* demonstrate significant variability depending on extraction methods, solvent polarity, plant parts, and processing conditions (Table 1 and Table 2). The diversity of phytochemical constituents identified across different studies contributes to a wide range of pharmacological effects. These effects include antioxidant, antidiabetic, antimicrobial, anticancer, anti-inflammatory, and cosmetic-related activities. Differences in total phenolic and flavonoid contents have been shown to influence the strength of biological activity [31]. The presence of specific compounds such as flavonoids, glycosides, and terpenoids further determines activity selectivity. Extraction methods such as maceration, Soxhlet, and decoction produce distinct phytochemical profiles. Solvent polarity also affects the distribution of bioactive compounds across fractions [32]. Fractionation and purification processes often enhance the biological potency of specific compounds. Advanced approaches such as nanoparticle synthesis further modify biological performance [33]. Endophytic fungi associated with the plant also contribute additional bioactive metabolites. Variations in plant origin and environmental conditions influence phytochemical accumulation [34]. Processing methods such as drying and fermentation can alter chemical composition. These variations lead to differences in biological activity outcomes across studies [35]. The interplay between extraction conditions and phytochemical composition plays a crucial role in shaping the observed pharmacological responses. Each biological activity reflects a specific combination of compounds and extraction parameters that determine its overall effectiveness.

### 3.1. Antioxidant Activity

Antioxidant activity was one of the most frequently reported biological effects of *Premna serratifolia*. The Kapuas Hulu maceration extract showed the strongest antioxidant activity, with an IC_50_ value of 18.39 mg/mL. This extract also had the highest total phenolic content, reaching 141.78 mg GAE/g. The Sambas Soxhlet extract contained the highest total flavonoid content at 15.23 mg QE/g [36]. However, the Sambas Soxhlet extract did not show the strongest antioxidant effect. This difference indicates that high flavonoid content did not always correspond to the best radical scavenging activity. Ethanol extracts prepared with increasing ethanol concentration also showed stronger antioxidant performance at higher ethanol percentages. The 100% ethanolic extract had the highest premnaodoroside A content, total phenolic content, and antioxidant activity expressed as 4.92–6.66% AAE [37]. In Central Sulawesi samples, the ethanol extract showed stronger DPPH scavenging than the water extract. The DPPH IC_50_ values were 50.63 ± 0.93 μg/mL for the ethanol extract and 66.83 ± 1.14 μg/mL for the water extract [1]. Decoction and infusion both exhibited strong antioxidant activity, with DPPH IC_50_ values of about 6.82 and 7.28 µg GAE/mL, respectively [38]. The decoction also showed stronger ferrous ion chelating activity than the infusion. Endophytic fungal extract from Aspergillus melleus demonstrated 63.91 ± 0.08% DPPH radical scavenging with an IC_50_ value of 96.14 µg/mL [39]. Silver nanoparticles synthesized from the leaf extract also showed strong antioxidant activity, including 73.5% DPPH scavenging and 69.24% hydrogen peroxide scavenging at 1000 µg/mL [40]. These data show that antioxidant activity varied according to extraction solvent, extraction method, formulation, and sample origin.

### 3.2. Antidiabetic Activity

Antidiabetic activity of *Premna serratifolia* was mainly assessed through α-glucosidase and α-amylase inhibition assays. The Sambas Soxhlet extract showed the strongest α-glucosidase inhibitory activity among the West Kalimantan samples, with an IC_50_ value of 4194.14 mg/mL reported [36]. In peatland samples from West Borneo, the ethyl acetate fraction had the strongest α-glucosidase inhibitory activity. This fraction reached 77.63 mg ACAE/100 g extract [41]. The ethanol fraction showed moderate inhibition at 55.89 mg ACAE/100 g extract. The hexane fraction showed lower inhibition at 39.74 mg ACAE/100 g extract. The water fraction showed no α-glucosidase inhibitory activity [42]. In Central Sulawesi samples, the ethanol extract inhibited α-glucosidase with an IC_50_ value of 151.91 ± 4.80 μg/mL. The same ethanol extract inhibited α-amylase with an IC_50_ value of 201.31 ± 2.43 μg/mL. The water extract showed weaker α-glucosidase inhibition and no α-amylase inhibition [1]. Decoction extract exhibited very strong α-glucosidase inhibition with an IC_50_ value of 0.046 µg GAE/mL. Infusion extract also showed strong inhibition with an IC_50_ value of 4.27 µg GAE/mL [38]. Endophytic fungal extract from Aspergillus melleus inhibited α-amylase and α-glucosidase with IC_50_ values of 181.41 and 190.62 µg/mL, respectively [39]. Molecular docking further showed that casticin, tricin, and centaureidin had strong binding interactions with digestive enzyme targets, supporting the in vitro inhibition results.

### 3.3. Antibacterial and Antimicrobial Activity

Antibacterial and antimicrobial activity of *Premna serratifolia* was observed in both plant-based and endophyte-derived systems. The Vietnamese aqueous leaf extract was used to synthesize silver nanoparticles for antibacterial testing. AgNP-coated silk showed inhibition greater than 99.78% against *Staphylococcus aureus*. The same material also showed inhibition greater than 99.99% against *Escherichia coli*. This antibacterial effect remained stable after 5–10 washing cycles [43]. Phytochemical analysis of the aqueous extract identified 18 compounds. Forsythoside E was the dominant compound, accounting for 32.14% of the extract. An endophytic fungus isolated from *Premna serratifolia*, identified as Hypocrea virens, also yielded potent antimicrobial compounds [43]. Two compounds were isolated, namely gliotoxin and bisdethiobis(methylthio)gliotoxin. Gliotoxin exhibited strong antimicrobial activity with an MIC of 0.13 µg/mL against *Bacillus subtilis*. Gliotoxin also inhibited *Staphylococcus aureus*, MRSA, *Escherichia coli*, *Pseudomonas aeruginosa*, and *Candida albicans*. In contrast, bisdethiobis(methylthio)gliotoxin showed weaker activity and was active only against *B. subtilis* [44]. Essential oil studies of fresh, dried, and fermented leaves identified volatile compounds such as linalool, phytol, methyl salicylate, and (E)-caryophyllene. Flower bud extracts also contained 94 volatile compounds, including 1-octen-3-ol, (Z)-3-hexenol, and 2-phenylethyl alcohol [45]. These results show that antimicrobial potential in *Premna serratifolia* was associated with nanoparticle formulations, endophytic metabolites, and volatile phytochemicals.

**Table 1:** Extraction Methods, Yield Characteristics, and Phytochemical Profiles of *Premna serratifolia*.

No	Plant Samples	Extraction Methods	Highest Extraction Yield	Quantitative Phytochemical Analysis	Results of Quantitative Phytochemical Analysis	Reference
1	Leaves of *Premna serratifolia* were collected from Sintang, Kapuas Hulu, and Sambas districts, West Kalimantan, Indonesia	Two extraction methods were applied, namely maceration and Soxhlet extraction, using ethanol as the solvent.	The highest extraction yield was obtained from the Soxhlet extraction of Sambas samples (SSI), reaching 23.011%.	Total phenolic content (TPC) was determined using the Folin–Ciocalteu method, while total flavonoid content (TFC) was measured using the AlCl_3_ method.	The highest TPC was observed in the Kapuas Hulu maceration extract (KHM) with a value of 141.78 mg GAE/g, while the highest TFC was found in the Sambas Soxhlet extract (SSI) with a value of 15.23 mg QE/g.	[36]
2	Leaves of *Premna serratifolia* (L.) were collected from Tan Thanh District, Long An Province, Vietnam	Aqueous extraction was performed using distilled water. Dried leaves (100 g) were extracted at 60–70 °C for 30 min, repeated three times, and the extract was concentrated to obtain the final product (PEAL).	A total of 33 g of aqueous extract was obtained from 100 g of dried leaves.	Phytochemical profiling was carried out using UPLC-QTOF-MS/MS to identify bioactive compounds present in the extract.	A total of 18 compounds were identified, including flavonoids, alkaloids, terpenoids, polyphenols, glycosides, and saponins. The dominant compound was Forsythoside E (32.14%), which plays a key role in reducing and stabilizing silver nanoparticles.	[43]
3	Leaves of *Premna serratifolia* L. were collected and used as the source for isolating the endophytic fungus Aspergillus melleus	The isolated endophytic fungus Aspergillus melleus was mass-cultured in liquid medium for 21 days, and its metabolites were extracted by maceration using methanol as the solvent. The extract was then concentrated using a rotary evaporator at 40 °C and 60 rpm.	Not available	Total phenolic content (TPC) was determined using the Folin–Ciocalteu reagent with gallic acid as the standard, while total flavonoid content (TFC) was measured using the aluminum chloride method with quercetin as the standard.	The methanolic extract of Aspergillus melleus showed a total phenolic content of 25.28 µg GAE/g and a total flavonoid content of 19.465 µg QE/g. Qualitative phytochemical screening also confirmed the presence of alkaloids, flavonoids, phenols, terpenoids, tannins, cardiac glycosides, and carbohydrates.	[39]
4	Leaves of *Premna serratifolia* Linn. (buas-buas) were used as the plant material in this study	The leaves were extracted using the maceration method with 96% ethanol as the solvent. The extraction was carried out for 3 × 24 h, followed by filtration and concentration using a rotary evaporator at 50 °C to obtain a thick extract.	The extraction yield obtained was 15.13%.	Not specifically performed in this study; however, the extract is known to contain secondary metabolites such as flavonoids, phenolics, alkaloids, saponins, tannins, and terpenoids.	Not quantitatively reported; previous studies cited indicate high flavonoid content and strong antioxidant activity of the extract.	[46]
5	Leaves of *Premna serratifolia* were used as the plant material in this study	The leaves were extracted and fractionated, and the ethyl acetate fraction was further isolated using column chromatography followed by semipreparative HPLC to obtain active subfractions.	Not available	Phytochemical profiling was carried out using UHPLC-Q-Orbitrap HRMS to identify flavonoid compounds present in the active ethyl acetate subfractions.	Nine flavonoids were identified, namely centaureidin, chrysin, pectolinaringenin, glycitein, kaempferide, syringetin, tricin, casticin, and 3,5,4′-trimethoxy-6,7-methylenedioxyflavone, the latter of which was predicted to be a new compound.	[47]
6	Leaves of *Premna serratifolia* and wood of Caesalpinia sappan were used as plant materials in this study.	Both plant materials were extracted using the maceration method with 96% ethanol as the solvent to obtain crude extracts.	Not available	Not quantitatively determined in this study; however, the extracts are known to contain phenolic compounds and flavonoids responsible for antioxidant activity.	Previous referenced studies indicated that *Premna serratifolia* contains flavonoids (4.67 mg/g; 0.47% *w*/*w*) and strong antioxidant-related phenolic compounds.	[48]
7	Leaves of *Premna serratifolia* L. were used as the plant material in this study	The leaves were extracted using the maceration method with 70% ethanol as the solvent, followed by concentration using a rotary evaporator to obtain a thick extract.	A yield of approximately 21.42% was obtained from 2100 g of simplicia, producing about 450 g of thick extract.	Not quantitatively determined in this study; however, the extract is known to contain secondary metabolites such as flavonoids, saponins, tannins, and triterpenoids/steroids.	Previous studies cited indicate that the extract possesses strong antioxidant-related flavonoid content, contributing to its bioactivity.	[41]
8	Leaves of *Premna serratifolia* L. were collected from three locations in Mandalay, Myanmar (Aungmyethazan, Chanayethazan, and Pathingyi)	The dried leaf powder was extracted using different ethanol concentrations (0%, 20%, 40%, 60%, 80%, and 100%) by heating at 100 °C for 2 h, followed by solvent evaporation to obtain crude extracts.	The highest extraction yield was obtained using 80% ethanol (up to 47.53%) and 40% ethanol (up to 45.96%), while 100% ethanol produced the lowest yield (13.57–19.57%).	Quantitative analysis was performed using a validated TLC-densitometric method to determine premnaodoroside A, along with total phenolic content (Folin–Ciocalteu method) and antioxidant activity (DPPH assay).	The highest premnaodoroside A content (3.23–5.25%), total phenolic content (7.56–8.24% GAE), and antioxidant activity (4.92–6.66% AAE) were observed in 100% ethanolic extracts, with values increasing proportionally to ethanol concentration.	[37]
9	Leaves of *Premna serratifolia* were collected from peatland areas near the Kapuas River, Pontianak, West Borneo, Indonesia	The dried leaves were macerated using ethanol for 3 days, followed by filtration and evaporation. The crude extract was then fractionated using liquid–liquid partition with solvents of different polarity: hexane, ethyl acetate, and water.	The highest extraction yield was obtained from the water fraction (30.63%), followed by ethanol (16.44%), ethyl acetate (10.21%), and hexane (7.74%).	Total flavonoid content (TFC), total phenolic content (TPC), and total steroid content (TSC) were determined using spectrophotometric methods, while functional groups were analyzed using FTIR spectroscopy.	The water fraction showed the highest TPC (21.84 g GAE/100 g extract) and TFC (6.56 g QE/100 g extract), while the hexane and ethyl acetate fractions exhibited relatively higher steroid content. Overall, phytochemical composition varied depending on solvent polarity.	[42]
10	Roots of *Premna serratifolia* L. were used as the plant material in this study. The roots were obtained from the Ayurvedic Garden, Department of Dravyagun, Faculty of Ayurveda, Institute of Medical Sciences, Banaras Hindu University, Varanasi, India	Fresh roots were washed, chopped into small pieces, and extracted with deionized water by heating 25 g of root material in 100 mL of water at 60 °C for 20 min. The extract was then filtered to obtain the aqueous extract of *Premna serratifolia* root (AEPS).	The aqueous root extract showed an extraction yield of 5.8%.	Phytochemical profiling was performed using UPLC-Q-TOF-MS/MS in both positive and negative ionization modes to tentatively identify the major compounds present in the aqueous root extract.	A total of 12 major polyphenolic compounds were tentatively identified, namely 4-hydroxy-3-methoxycinnamic acid, linarin, peonidin-3,5-O-di-beta-glucopyranoside, diosmin, trans-cinnamic acid, daidzein, saponarin, homoorietin, acacetin, sarsasapogenin, phytol, and sissotrin. Eleven of these compounds were reported for the first time from the aqueous root extract, while phytol had been previously reported in the root.	[16]
11	Leaves of *Premna serratifolia* L. were collected from Mandalay Division, Myanmar. The samples were divided into fresh, dried, and fermented leaves for comparative analysis.	Essential oils were obtained using hydrodistillation with a Clevenger apparatus for 5 h. Fresh, dried, and fermented leaves were processed separately, and the oils were dried over anhydrous sodium sulfate and stored at 4 °C.	The essential oil yields were 0.008% (fresh leaves), 0.139% (dried leaves), and 0.139% (fermented leaves), indicating higher yield after drying and fermentation.	Chemical profiling was performed using GC-MS with DB-5 and Carbowax 20 M columns. Compound identification was based on retention indices and comparison with spectral databases.	A total of 77, 82, and 90 compounds were identified in fresh, dried, and fermented leaves, respectively. Major compound classes included hydrocarbons, terpenoids, and phenolics. The dominant compounds in fresh leaves were amyl vinyl carbinol (15.8–32.6%), linalool (11.1–15.1%), phytol (7.7–12.5%), methyl salicylate (3.9–7.2%), and (E)-caryophyllene (3.1–6.6%). Drying and fermentation significantly altered chemical composition, including the decrease in linalool and phytol, the increase of (E)-caryophyllene, and the formation of new compounds such as β-myrcene. Fermentation notably increased phenolic compounds, especially p-vinyl anisole (up to 41.1%), and introduced acorenone B as a new compound.	[49]
12	Leaves of *Premna serratifolia* L. were collected from Tentena, Poso, Central Sulawesi, Indonesia, and used as the plant material in this study	Two extracts were prepared from the dried leaf powder: an ethanol extract obtained by maceration in ethanol at room temperature for 3 days, and a water extract obtained by decoction in deionized water at 90 °C until the volume was reduced by half, followed by freeze-drying.	Not available	Total phenolic content (TPC) was determined using the Folin–Ciocalteu method, total flavonoid content (TFC) was measured by the aluminum chloride colorimetric method, and phytochemical profiling of the ethanol extract was performed using LC-QTOF-MS/MS.	The ethanol extract showed higher total phenolic content than the water extract, with values of 2.12 ± 0.06 and 0.27 ± 0.00 mg GAE/g dried biomass, respectively. In contrast, the water extract had a higher total flavonoid content than the ethanol extract, with values of 12.11 ± 0.20 and 9.43 ± 0.04 mg RE/g dried biomass, respectively. LC-QTOF-MS/MS analysis identified seven major compounds in the ethanol extract, namely scroside E, forsythoside A, forsythoside B, lavandulifolioside, diosmin, nobilin D, campneoside I, and isoacteoside.	[1]
13	Stem wood of *Premna serratifolia* was collected from Myeik Township, Tanintharyi Region, Myanmar, and used as the plant material in this study	The dried powdered stem wood (4.8 kg) was extracted by sonication using chloroform (8 L, 90 min, repeated four times). The extract was then concentrated and subjected to silica gel column chromatography, followed by further purification using HPLC and preparative TLC.	A total of 31.5 g of chloroform extract was obtained from 4.8 kg of dried stem wood.	Structural elucidation of compounds was performed using spectroscopic techniques including ^1^H NMR, ^13^C NMR, IR spectroscopy, UV spectroscopy, and high-resolution electrospray ionization mass spectrometry (HRESIMS), supported by circular dichroism (CD) and DFT/TDDFT calculations.	Eight lignoid compounds were identified, including four new lignoids (premnan A, premnan B, taungtangyiol C, and premnan C), one naturally occurring new lignoid, and three known lignoids. The structures were confirmed through detailed spectroscopic and computational analysis.	[50]
14	Stem wood of *Premna serratifolia* (syn. *Premna integrifolia*) was collected from Myeik Township, Tanintharyi Region, Myanmar, and used as the plant material in this study	The dried stem wood (4.8 kg) was powdered and extracted using chloroform (CHCl_3_) by sonication (8 L, 90 min, repeated four times) at room temperature. The extract was then concentrated and subjected to repeated silica gel column chromatography and preparative TLC for compound isolation.	A total of 31.5 g of chloroform extract was obtained from 4.8 kg of dried stem wood.	Structural elucidation was performed using spectroscopic techniques including ^1^H NMR, ^13^C NMR, IR, UV, and high-resolution electrospray ionization mass spectrometry (HRESIMS), supported by COSY, HMBC, and NOESY analyses.	One new tetrahydrofuran-type lignan, 7,9-dihydroxydolichanthin B (1), and two known triterpenoids, oleanonic acid (2) and (2α,3α)-dihydroxyolean-12-en-28-oic acid (3), were successfully isolated and structurally characterized.	[51]
15	Leaves of *Premna serratifolia* L. were collected from Tentena, Poso, Central Sulawesi, Indonesia, and used as the plant material in this study	Aqueous extracts were prepared using two traditional methods: infusion and decoction. The infusion was prepared by immersing 2 g of leaf powder in 200 mL of boiling water and allowing it to cool for 15 min, while the decoction was prepared by boiling 2 g of leaf powder in 200 mL of water until the volume was reduced to 100 mL.	Not available	Total phenolic content (TPC) was determined using the Folin–Ciocalteu method, while chemical profiling was performed using UV-Vis spectrophotometry and LC-MS analysis.	The decoction showed higher total phenolic content (539.26 ± 7.44 µg GAE/mL) compared to the infusion (347.81 ± 0.21 µg GAE/mL). UV spectra indicated the presence of phenolic compounds, and LC-MS analysis revealed a dominant peak at *m*/*z* 132, tentatively identified as caffeic acid.	[38]
16	Leaves of *Premna serratifolia* were collected from the Regional Medical Research Centre (ICMR), Belagavi, India, and authenticated with a voucher specimen (RMRC-554)	The dried powdered leaves were extracted using methanol, followed by fractionation. The n-hexane soluble fraction was subjected to saponification to obtain the unsaponifiable matter, which was further separated using silica gel column chromatography and preparative TLC to isolate active compounds.	Not available	Phytochemical identification was performed using TLC, HR-GCMS, HR-LCMS, and NMR (^1^H and ^13^C), along with chemical tests such as the Liebermann–Burchard reaction to confirm terpenoids and steroids.	Three main compounds were isolated, namely oleanolic acid (PS-01A), stigmasterol (PS-01B), and an unknown compound (PS-02A). Spectroscopic analysis confirmed the structures of the known compounds, while LC-MS revealed that PS-02A contained multiple components with *m*/*z* values of 573.25, 387.18, and 415.21.	[52]
17	Leaves and twigs of *Premna serratifolia* were collected from the Kadol Kale mangrove forest in Negombo Lagoon, Sri Lanka, and used for the isolation of endophytic fungi	Endophytic fungi were isolated through surface sterilization of plant tissues followed by culturing on potato dextrose agar (PDA). The fungal cultures were extracted using ethyl acetate, and the crude extract of the most active strain was subjected to bioassay-guided fractionation using Sephadex LH-20 column chromatography and silica gel column chromatography to isolate bioactive compounds.	Not available	Structural elucidation of isolated compounds was performed using spectroscopic techniques including ^1^H NMR, ^13^C NMR, 2D NMR (COSY, HSQC, HMBC, TROESY), and electrospray ionization mass spectrometry (ESIMS).	Two major compounds were isolated from the endophytic fungus Hypocrea virens, namely gliotoxin and bisdethiobis(methylthio)gliotoxin. Structural analysis confirmed that gliotoxin possesses an epidithiodioxopiperazine core, while the second compound is a methylated derivative with additional S-methyl groups.	[44]
18	Leaves of *Premna serratifolia* were collected from Megamalai, Varusanadu Hills, Theni District, Tamil Nadu, India, and used for nanoparticle synthesis	The dried leaf powder (100 g) was extracted using ethanol by Soxhlet extraction (600 mL, 48 h). The extract was then used for the biosynthesis of silver nanoparticles by reacting with a 1 mM AgNO_3_ solution, resulting in the formation of AgNPs indicated by a color change to yellowish brown.	Not available	Characterization of the synthesized nanoparticles was performed using UV–Vis spectroscopy, SEM, FTIR, and XRD analysis. The Debye–Scherrer equation was applied to determine particle size.	UV–Vis analysis showed a maximum absorption peak at ~460 nm, confirming nanoparticle formation. SEM analysis revealed particle sizes ranging from 15–100 nm, while XRD analysis confirmed a face-centered cubic structure with an average particle size of 22.97 nm. FTIR results indicated the involvement of proteins and functional groups (OH, amide, C–N) acting as reducing and stabilizing agents in nanoparticle formation.	[40]
19	Leaves of *Premna serratifolia* L. were collected from Universiti Putra Malaysia, Bintulu Sarawak Campus, at different times of the day (6 am, 9 am, 12 noon, 3 pm, and 6 pm) to evaluate environmental effects on phytochemical content	The leaves were washed, oven-dried at 50 °C for two days, and extracted using hot water (60 °C for 1 h). The extract was then filtered and concentrated using a rotary evaporator below 40 °C.	Not available	Total phenolic content (TPC) was determined using the Folin–Ciocalteu method, total flavonoid content (TFC) using the aluminum chloride method, and antioxidant activity was measured using the DPPH radical scavenging assay. Environmental parameters (light intensity and temperature) were also recorded and correlated with phytochemical content.	The highest total phenolic content (590 µg pyrocatechol equivalent/mg extract) and total flavonoid content (883 µg quercetin equivalent/mg extract) were observed in leaves collected at 12 noon, while the lowest values were found in leaves collected at 9 am. Both phenolic and flavonoid contents showed a positive correlation with temperature and light intensity.	[53]
20	Leaves of *Premna serratifolia* were collected from the Regional Medical Research Centre (ICMR), Belgaum, India, and authenticated with a voucher specimen (RMRC-554)	The leaves were extracted using methanol, followed by fractionation to obtain the n-hexane soluble fraction. The unsaponifiable portion of this fraction was subjected to silica gel column chromatography using gradient elution (petroleum ether, chloroform, and acetone), yielding five sub-fractions (PS-01 to PS-05)	Not available	Phytochemical analysis was performed using TLC and standard chemical tests. Optimization of the TLC system was carried out using toluene:ethyl acetate:glacial acetic acid (7:2:1), and all sub-fractions were identified as triterpenoids based on chromatographic and chemical profiling.	Five sub-fractions (PS-01 to PS-05) were obtained, with PS-01 and PS-02 identified as single-compound fractions. TLC analysis confirmed distinct Rf values (e.g., PS-02 at 0.65), and all fractions were characterized as triterpenoids.	[54]
21	Leaves, root bark (RB), and root wood (RW) of *Premna serratifolia* were collected from Kerala, India, and used as plant materials for phytochemical and biological evaluation	The dried plant materials (leaves, RB, and RW) were extracted using methanol by soaking for 2 weeks. The extracts were then subjected to solvent–solvent partitioning with petroleum ether, chloroform, and ethyl acetate. The active chloroform fraction (from RB) was further purified using Sephadex LH-20 column chromatography, Combiflash chromatography, and preparative HPLC to isolate the bioactive compound.	Methanolic extraction yielded approximately 12 g (leaves), 11 g (root bark), and 10 g (root wood) from 250–280 g of dried plant material	Structural elucidation of the isolated compound was performed using advanced spectroscopic techniques including ^1^H NMR, ^13^C NMR, and 2D-NMR (COSY, HMQC, HMBC, NOESY), as well as mass spectrometry (ESI-MS) and HPLC analysis.	A novel diterpene compound, identified as 11,12,16-trihydroxy-2-oxo-5-methyl-10-demethyl-abieta-type diterpene, was successfully isolated from the root bark extract. The compound exhibited a unique C-5 methyl abietane-type skeleton with extended aromatic features, confirmed through detailed NMR correlations and MS data.	[55]
22	Fresh flower buds of *Premna serratifolia* were collected from Tautira district, Tahiti Island, French Polynesia, during the dry season	The flower buds were extracted using hexane at room temperature (30 °C) with sonication for 20 min to obtain a concentrate. The extract was then concentrated and subjected to vacuum distillation (up to 120 °C/0.05 mmHg) to obtain the volatile fraction. Further fractionation was performed using silica gel column chromatography with increasing polarity solvents (hexane, diethyl ether, methanol).	The concrete yield was approximately 0.3%, and the volatile fraction represented about 30% of the distilled material.	Chemical composition of the volatile fraction was analyzed using GC and GC-MS, with compound identification based on retention indices and comparison with spectral libraries.	A total of 94 volatile compounds were identified, representing approximately 81% of the distillate (see Table 1 on page 4). The major constituents included 1-octen-3-ol (16.9%), (Z)-3-hexenol (10.2%), 2-phenylethyl alcohol (8.9%), (E,Z)-2,4-nonadienal (6.2%), (E,Z)-2,6-nonadienal (5.0%), and linalool (4.4%).	[45]

**Table 2:** Biological Activities of *Premna serratifolia* Extracts, Fractions, and Related Preparations.

No	Plant Samples	Biological Activity Assay	Results of Biological Activity Assay	Reference
1	Leaves of *Premna serratifolia* were collected from Sintang, Kapuas Hulu, and Sambas districts, West Kalimantan, Indonesia	Biological activities were evaluated through antioxidant activity using the DPPH method and α-glucosidase inhibitory activity using the PNPG method.	The strongest antioxidant activity was exhibited by the KHM with an IC_50_ value of 18.39 mg/mL, while the strongest α-glucosidase inhibitory activity was shown by the SSI with an IC_50_ value of 4194.14 mg/mL.	[36]
2	Leaves of *Premna serratifolia* (L.) were collected from Tan Thanh District, Long An Province, Vietnam	Biological activity was evaluated through antibacterial testing against *Staphylococcus aureus* and *Escherichia coli* using a shaking flask method.	AgNPs-coated silk exhibited excellent antibacterial activity, showing inhibition greater than 99.78% against *S. aureus* and greater than 99.99% against *E. coli*. The antibacterial effect remained stable even after 5–10 washing cycles, indicating strong durability.	[43]
3	Leaves of *Premna serratifolia* L. were collected and used as the source for isolating the endophytic fungus Aspergillus melleus	Biological activities were evaluated through antioxidant assays including phosphomolybdenum, FRAP, and DPPH radical scavenging assays; antidiabetic assays using α-amylase and α-glucosidase inhibition methods; and anti-inflammatory assays using protein denaturation, COX-1, and COX-2 inhibition methods.	The extract exhibited 63.91 ± 0.08% DPPH radical scavenging activity with an IC_50_ value of 96.14 µg/mL. In antidiabetic testing, the IC_50_ values were 181.41 µg/mL for α-amylase inhibition and 190.62 µg/mL for α-glucosidase inhibition. In anti-inflammatory testing, the extract showed strong protein denaturation inhibition and COX inhibitory activity, with IC_50_ values of 118.20 µg/mL for protein denaturation, 68.53 µg/mL for COX-1, and 43.34 µg/mL for COX-2.	[39]
4	Leaves of *Premna serratifolia* Linn. (buas-buas) were used as the plant material in this study	Biological activity was evaluated through sunscreen activity testing using UV-Vis spectrophotometry (290–320 nm) and calculation of Sun Protection Factor (SPF).	The lotion formulations showed increasing sunscreen activity with higher extract concentration. The SPF values were 7.12 (1%, extra protection), 9.54 (2%, maximum protection), and 15.68 (3%, ultra protection), indicating effective UV protection potential.	[46]
5	Leaves of *Premna serratifolia* were used as the plant material in this study	Biological activity was evaluated through α-glucosidase inhibitory assay in vitro, followed by molecular docking analysis against N-terminal maltase-glucoamylase (2QMJ), C-terminal maltase-glucoamylase (3TOP), and isomaltase (3A4A).	The F14 subfraction showed the best α-glucosidase inhibitory activity. Molecular docking revealed that casticin showed the strongest interaction with 2QMJ with a binding energy of −5.29 kcal/mol and Ki of 131.54 µM, tricin showed the strongest interaction with 3TOP with a binding energy of −6.77 kcal/mol and Ki of 10.89 µM, and centaureidin showed the strongest interaction with 3A4A with a binding energy of −8.02 kcal/mol and Ki of 0.34 µM.	[47]
6	Leaves of *Premna serratifolia* and wood of Caesalpinia sappan were used as plant materials in this study.	Biological activity was evaluated through antioxidant activity using the DPPH method for both crude extracts and gel formulations.	The crude extract combination showed very strong antioxidant activity with IC_50_ values of 0.0329 mg/mL (F1), 0.0246 mg/mL (F2), and 0.2282 mg/mL (F3). In gel formulations, the IC_50_ values were 3.8677 mg/mL (F1), 4.3953 mg/mL (F2), and 4.396 mg/mL (F3), indicating reduced but still notable antioxidant activity after formulation.	[48]
7	Leaves of *Premna serratifolia* L. were used as the plant material in this study	Biological activity was evaluated through antioxidant activity using the DPPH method, performed on peel-off gel mask formulations before and after stability (cycling) tests.	The peel-off gel mask showed antioxidant activity with percent inhibition ranging from 51.76% to 77.63%. The highest activity was observed in Formula III (3% extract) with an average inhibition of 77.20 ± 0.28%, and no significant change in activity was observed after stability testing.	[41]
8	Leaves of *Premna serratifolia* L. were collected from three locations in Mandalay, Myanmar (Aungmyethazan, Chanayethazan, and Pathingyi)	Biological activity was evaluated through antioxidant activity using the DPPH radical scavenging method.	Antioxidant activity increased with higher ethanol concentration, with the 100% ethanolic extract showing the strongest activity, expressed as 4.92–6.66% ascorbic acid equivalent (AAE).	[37]
9	Leaves of *Premna serratifolia* were collected from peatland areas near the Kapuas River, Pontianak, West Borneo, Indonesia	Biological activity was evaluated through α-glucosidase inhibitory activity using the PNPG method, with acarbose as a reference standard.	The ethyl acetate fraction showed the strongest α-glucosidase inhibitory activity (77.63 mg ACAE/100 g extract), followed by ethanol (55.89 mg ACAE/100 g extract) and hexane (39.74 mg ACAE/100 g extract), while the water fraction showed no activity. Multivariate analysis indicated that flavonoids (TFC) and H–C=O aldehyde functional groups were the main contributors to the inhibitory activity.	[42]
10	Roots of *Premna serratifolia* L. were used as the plant material in this study. The roots were obtained from the Ayurvedic Garden, Department of Dravyagun, Faculty of Ayurveda, Institute of Medical Sciences, Banaras Hindu University, Varanasi, India	Biological activity was evaluated through cytotoxicity testing against the human hepatoblastoma cell line Hep G2 using MTT assay, supported by Hoechst staining, AO/EtBr staining, ROS measurement, mitochondrial membrane potential analysis, clonogenic assay, and wound healing assay.	The aqueous root extract exhibited cytotoxic activity against Hep G2 cells with an IC_50_ value of 1000 µg/mL after 48 h of incubation. The extract induced dose- and time-dependent cell death, increased intracellular ROS, reduced mitochondrial membrane potential, inhibited colony formation and wound healing, and promoted apoptotic changes in Hep G2 cells, while remaining non-cytotoxic to primary rat hepatocytes up to the same concentration.	[16]
11	Leaves of *Premna serratifolia* L. were collected from Mandalay Division, Myanmar. The samples were divided into fresh, dried, and fermented leaves for comparative analysis.	No direct biological activity assays were conducted in this study; however, biological activities were discussed based on previously reported data of the identified compounds.	Major compounds such as linalool, (E)-caryophyllene, phytol, β-myrcene, and methyl salicylate are known to exhibit antimicrobial, antioxidant, anti-inflammatory, antiproliferative, and anticancer activities. Changes in chemical composition due to drying and fermentation suggest potential variation in biological activity among fresh, dried, and fermented leaves.	[49]
12	Leaves of *Premna serratifolia* L. were collected from Tentena, Poso, Central Sulawesi, Indonesia, and used as the plant material in this study	Biological activity was evaluated through antioxidant assays including DPPH radical scavenging, CuPRAC, phosphomolybdenum, reducing power, and DNA protection assay, as well as enzyme inhibition assays against α-glucosidase, α-amylase, xanthine oxidase, and protease.	The ethanol extract demonstrated stronger antioxidant activity than the water extract, with DPPH IC_50_ values of 50.63 ± 0.93 and 66.83 ± 1.14 μg/mL, respectively. The ethanol extract also showed stronger inhibition of α-glucosidase and α-amylase, with IC_50_ values of 151.91 ± 4.80 and 201.31 ± 2.43 μg/mL, respectively, while the water extract inhibited α-glucosidase more weakly and showed no α-amylase inhibition. Both extracts showed weak inhibition of xanthine oxidase and protease, although the ethanol extract remained more active than the water extract. In addition, the ethanol extract exhibited a concentration-dependent protective effect against hydroxyl radical-induced DNA damage.	[1]
13	Stem wood of *Premna serratifolia* was collected from Myeik Township, Tanintharyi Region, Myanmar, and used as the plant material in this study	Biological activity was evaluated using melanogenesis assays in B16-F10 mouse melanoma cells, with α-MSH and IBMX as inducers.	The chloroform extract exhibited anti-melanin deposition activity with an IC_50_ value of 81.3 µg/mL. In contrast, isolated compounds (1–4 and 6) showed melanogenesis-enhancing activity. Compounds 1 and 4 increased melanin production by 31% and 50% at 50 µM, respectively, while compounds 2, 3, and 6 increased melanin production by 67%, 30%, and 45% at 100 µM, without significant cytotoxicity at moderate concentrations	[50]
14	Stem wood of *Premna serratifolia* (syn. P. integrifolia) was collected from Myeik Township, Tanintharyi Region, Myanmar, and used as the plant material in this study	Biological activity was evaluated through anti-melanin deposition assays using B16-F10 mouse melanoma cells induced by α-MSH and IBMX.	All isolated compounds exhibited strong anti-melanin deposition activity, with IC_50_ values of 18.4 µM (compound 1), 17.7 µM (compound 2), and 11.2 µM (compound 3), which were significantly more potent than the positive control arbutin (IC_50_ = 698 µM). Compound 2 showed slight cytotoxicity at concentrations above 50 µM, while compounds 1 and 3 did not exhibit cytotoxic effects even at higher concentrations.	[51]
15	Leaves of *Premna serratifolia* L. were collected from Tentena, Poso, Central Sulawesi, Indonesia, and used as the plant material in this study	Biological activity was evaluated through α-glucosidase inhibitory assay, DPPH radical scavenging assay, and ferrous ion chelating activity assay.	Both infusion and decoction extracts exhibited strong α-glucosidase inhibitory activity, with IC_50_ values of 4.27 µg GAE/mL (infusion) and 0.046 µg GAE/mL (decoction), which were significantly more potent than acarbose. In antioxidant testing, both extracts showed strong DPPH scavenging activity (IC_50_ ≈ 6.82 and 7.28 µg GAE/mL for infusion and decoction, respectively), while decoction demonstrated stronger ferrous ion chelating activity (IC_50_ = 28.34 µg GAE/mL) compared to infusion (IC_50_ = 161.66 µg GAE/mL)	[38]
16	Leaves of *Premna serratifolia* were collected from the Regional Medical Research Centre (ICMR), Belagavi, India, and authenticated with a voucher specimen (RMRC-554)	Biological activity was evaluated through cytotoxicity testing using brine shrimp lethality (BSL) assay and MTT assay on cancer cell lines A549 (lung cancer), HepG2 (liver cancer), and L6 (normal cell line).	All isolated compounds exhibited cytotoxic activity. In the BSL assay, LC_50_ values were 54.49 ppm (oleanolic acid), 30.83 ppm (stigmasterol), and 16.32 ppm (PS-02A), indicating strong toxicity. In cell line studies, PS-02A showed the highest cytotoxicity with IC_50_ values of 66.77 µg/mL (A549) and 53.72 µg/mL (HepG2), while showing lower toxicity toward normal L6 cells (IC_50_ = 112.93 µg/mL), suggesting selective anticancer potential.	[52]
17	Leaves and twigs of *Premna serratifolia* were collected from the Kadol Kale mangrove forest in Negombo Lagoon, Sri Lanka, and used for the isolation of endophytic fungi	Biological activity was evaluated through antimicrobial assays using agar disc diffusion and broth microdilution methods against Gram-positive bacteria (*Bacillus subtilis*, *Staphylococcus aureus*, MRSA), Gram-negative bacteria (*Escherichia coli*, *Pseudomonas aeruginosa*), and the fungus *Candida albicans*.	Gliotoxin exhibited strong antimicrobial activity, with MIC values of 0.13 µg/mL against *B. subtilis*, 16 µg/mL against *S. aureus*, 32 µg/mL against MRSA and *E. coli*, and 64 µg/mL against *P. aeruginosa* and *C. albicans*. In contrast, bisdethiobis(methylthio)gliotoxin showed weaker activity and was only active against *B. subtilis* with moderate inhibition. These results indicate that gliotoxin is the primary bioactive antimicrobial compound produced by the endophytic fungus.	[44]
18	Leaves of *Premna serratifolia* were collected from Megamalai, Varusanadu Hills, Theni District, Tamil Nadu, India, and used for nanoparticle synthesis	Anticancer activity was evaluated using a CCl_4_-induced liver cancer model in Swiss albino mice, along with biochemical assays including SGOT, SGPT, total protein, TBARS, DPPH, and hydrogen peroxide scavenging assays.	Silver nanoparticles coated with *Premna serratifolia* extract significantly improved biochemical parameters compared to untreated cancer-induced mice, restoring SGOT, SGPT, total protein, and TBARS levels closer to normal. The AgNP-treated group showed better recovery than plant extract alone or isoleucine treatment. Additionally, strong antioxidant activity was observed, with up to 73.5% DPPH scavenging and 69.24% hydrogen peroxide scavenging at 1000 µg/mL. These findings indicate that biosynthesized AgNPs exhibit enhanced anticancer and antioxidant activity compared to the crude extract.	[40]
19	Leaves of *Premna serratifolia* L. were collected from Universiti Putra Malaysia, Bintulu Sarawak Campus, at different times of the day (6 am, 9 am, 12 noon, 3 pm, and 6 pm) to evaluate environmental effects on phytochemical content	Biological activity was evaluated through antioxidant activity using the DPPH radical scavenging assay, along with correlation analysis between antioxidant activity, phenolic content, flavonoid content, light intensity, and temperature.	The highest DPPH antioxidant activity (73.5%) was observed in leaves collected at 9 am, while the lowest activity (~47.7%) occurred at 12 noon. Antioxidant activity showed a negative correlation with phenolic and flavonoid content, as well as with temperature and light intensity, indicating that higher environmental stress conditions increased phytochemical accumulation but reduced radical scavenging efficiency.	[53]
20	Leaves of *Premna serratifolia* were collected from the Regional Medical Research Centre (ICMR), Belgaum, India, and authenticated with a voucher specimen (RMRC-554)	Biological activity was evaluated using the brine shrimp lethality (BSL) assay for initial screening, followed by in vitro cytotoxicity testing using the MTT assay on human cancer cell lines MCF-7 (breast carcinoma) and HT-29 (colorectal adenocarcinoma)	The BSL assay showed that PS-01 and PS-02 were the most active fractions, with LC_50_ values of 54.6 µg/mL and 30.8 µg/mL, respectively. Further testing revealed that PS-02 exhibited significant cytotoxicity against both MCF-7 and HT-29 cell lines, with LC_50_ values of approximately 100.0 µg/mL and 99.9 µg/mL, respectively, indicating the strong anticancer potential of triterpenoid constituents.	[54]
21	Leaves, root bark (RB), and root wood (RW) of *Premna serratifolia* were collected from Kerala, India, and used as plant materials for phytochemical and biological evaluation	Biological activity was evaluated through cytotoxicity assays against SHSY-5Y neuroblastoma cells and B16 melanoma cells using the Alamar Blue assay, as well as antioxidant activity using the DPPH radical scavenging assay	The isolated diterpene showed strong cytotoxic activity, with IC_50_ values of 1.5 µg/mL (SHSY-5Y) and 4.7 µg/mL (B16), which were approximately 21–23 times more potent than the crude extract. Additionally, the compound exhibited antioxidant activity with a DPPH IC_50_ value of 20.4 ± 1.3 µM, comparable to caffeic acid (IC_50_ = 14.4 ± 1.6 µM). These findings indicate that the novel diterpene possesses both anticancer and antioxidant potential.	[55]
22	Fresh flower buds of *Premna serratifolia* were collected from Tautira district, Tahiti Island, French Polynesia, during the dry season	Not available	Not applicable; however, the identified volatile compounds (e.g., linalool, phenylethyl alcohol, and aldehydes) are known from literature to contribute to fragrance, antimicrobial, and antioxidant properties.	[45]

### 3.4. Anticancer and Cytotoxic Activity

Cytotoxic and anticancer activities were reported from leaves, roots, root bark, and derived fractions of *Premna serratifolia*. The aqueous root extract showed cytotoxicity against Hep G2 cells with an IC_50_ value of 1000 µg/mL after 48 h [16]. This extract induced dose- and time-dependent cell death. It also increased intracellular ROS and reduced mitochondrial membrane potential. In addition, it inhibited colony formation and wound healing in Hep G2 cells. Methanolic leaf extract fractionation yielded compounds such as oleanolic acid, stigmasterol, and PS-02A. In the brine shrimp lethality assay, PS-02A showed the highest toxicity with an LC_50_ value of 16.32 ppm. In cell line studies, PS-02A showed IC_50_ values of 66.77 µg/mL against A549 and 53.72 µg/mL against HepG2. This fraction showed lower toxicity toward normal L6 cells, with an IC_50_ value of 112.93 µg/mL [52]. Another fractionation study using MCF-7 and HT-29 cells showed that PS-02 was the most active fraction. Its LC_50_ values were approximately 100.0 µg/mL against MCF-7 and 99.9 µg/mL against HT-29 [54]. A novel diterpene isolated from root bark exhibited much stronger cytotoxicity. This compound showed IC_50_ values of 1.5 µg/mL against SHSY-5Y cells and 4.7 µg/mL against B16 cells [55]. These values were 21–23 times more potent than the crude root bark extract. The data indicate that purified compounds from *Premna serratifolia* generally showed stronger anticancer activity than crude extracts.

### 3.5. Anti-Inflammatory, Cosmetic, and Other Functional Activities

Anti-inflammatory, cosmetic, and other functional activities were also reported for *Premna serratifolia*. The endophytic fungal extract from Aspergillus melleus showed measurable anti-inflammatory effects. Its IC_50_ value was 118.20 µg/mL for protein denaturation inhibition. The same extract inhibited COX-1 with an IC_50_ value of 68.53 µg/mL. It also inhibited COX-2 with an IC_50_ value of 43.34 µg/mL [39]. Sunscreen activity was demonstrated in lotion formulations prepared from leaf extract. The SPF value increased from 7.12 at 1% extract to 9.54 at 2% extract. At 3% extract concentration, the SPF value reached 15.68 [46]. These results indicate concentration-dependent enhancement of UV protection. Cosmetic relevance was also observed in melanogenesis studies of stem wood extracts and isolated compounds. The chloroform extract showed anti-melanin deposition activity with an IC_50_ value of 81.3 µg/mL. In contrast, several isolated lignoid compounds enhanced melanin production by 30% to 67%, depending on the compound and concentration [50]. Another study identified a new tetrahydrofuran-type lignan and two triterpenoids with anti-melanin deposition activity stronger than arbutin [51]. Essential oil studies further showed that drying and fermentation altered chemical composition, increasing the number of identified compounds from 77 in fresh leaves to 90 in fermented leaves. These findings indicate that *Premna serratifolia* has multifunctional properties that extend beyond classic pharmacological activities.

## 4. Phytochemical Profiling of *Premna serratifolia*

*Premna serratifolia* is a medicinal plant widely distributed in tropical regions and has long been used in traditional medicine for treating inflammation, infections, metabolic disorders, and skin-related conditions [56,57]. Recent studies have reported that this plant contains a diverse range of secondary metabolites, including flavonoids, iridoid glycosides, phenylpropanoid glycosides, terpenoids, and phenolic compounds. These metabolites are responsible for various pharmacological activities such as antioxidant, antidiabetic, antimicrobial, anticancer, and anti-inflammatory effects [58,59]. Advances in analytical techniques such as LC–MS and UPLC-Q-Orbitrap HRMS have enabled detailed identification of these compounds. The identified metabolites show significant structural diversity, ranging from simple phenolic acids to complex glycosylated molecules. Differences in chemical structure influence physicochemical properties and biological activity [60]. In silico approaches such as SwissADME analysis provide valuable insights into drug-likeness and pharmacokinetic behavior of these compounds. Evaluation based on Lipinski’s rule and lead-likeness criteria helps determine the suitability of compounds as potential drug candidates [61]. Compounds with favorable physicochemical properties are more likely to exhibit good absorption and bioavailability. In contrast, highly glycosylated compounds often show limitations due to large molecular size and high polarity [62]. The relationship between chemical structure and pharmacokinetic properties is essential for understanding drug development potential. Table 3 and Table 4 summarize the identified compounds from *Premna serratifolia* along with their chemical classification and molecular characteristics. Table 5 presents the drug-likeness, lead-likeness, and synthetic accessibility profiles of selected compounds based on in silico analysis. These datasets provide a comprehensive overview of the chemical diversity and pharmacokinetic potential of the plant. The integration of phytochemical identification and ADMET-related evaluation supports the selection of promising compounds for further pharmacological and computational studies.

### 4.1. Flavonoids (Flavone, Flavonol, Isoflavone, and Their Glycosides)

Flavonoids represent the most abundant class of compounds identified from *Premna serratifolia* based on LC–MS and UPLC-Q-Orbitrap HRMS profiling. This class includes flavone, flavonol, isoflavone, and their glycoside derivatives such as centaureidin, chrysin, tricin, luteolin, acacetin, and daidzein. Most flavonoid aglycones showed molecular weights below 350 g/mol and satisfied Lipinski’s rule without violations. These compounds also exhibited balanced physicochemical properties, including moderate lipophilicity and hydrogen bonding capacity [63]. Several compounds such as glycitein, kaempferide, pectolinarigenin, tricin, luteolin, acacetin, and daidzein fulfilled lead-likeness criteria. Synthetic accessibility scores for these compounds ranged between 2.79 and 3.41, indicating relatively simple structures [64]. Polymethoxylated flavones such as centaureidin and casticin exhibited slightly higher molecular weights of 360.3 and 374.3 g/mol, leading to minor lead-likeness violations. These compounds also showed moderate synthetic accessibility values above 3.5. Glycosylated flavonoids such as diosmin, isorhamnetin-3-O-glucoside, kaempferol derivatives, linarin, saponarin, and homoorientin showed molecular weights exceeding 500 g/mol. These compounds exhibited multiple Lipinski violations related to hydrogen bond donors and acceptors. Their synthetic accessibility scores were higher, ranging from 5.17 to 6.48, indicating increased structural complexity. Anthocyanin glycosides such as peonidin-3,5-O-di-glucopyranoside also showed high molecular weight and multiple violations. Pharmacokinetic radar profiles indicated that glycosylated flavonoids exceed optimal polarity and size ranges. These properties limit passive membrane diffusion and oral absorption [65]. Aglycone flavonoids displayed more favorable pharmacokinetic profiles with better compliance to drug-likeness criteria. The data indicate that structural simplicity and absence of sugar moieties improve drug-likeness within this class.

### 4.2. Iridoid Glycosides

Iridoid glycosides identified from *Premna serratifolia* include catalpol derivatives and premnaodoroside B with complex glycosylated structures. These compounds exhibited molecular weights ranging from 508.47 to 890.96 g/mol. Most iridoid glycosides showed three Lipinski violations related to molecular weight, hydrogen bond donors, and hydrogen bond acceptors. These violations indicate high polarity and limited membrane permeability [66]. Lead-likeness analysis showed additional violations associated with molecular weight and rotatable bonds exceeding seven. Synthetic accessibility scores ranged from 6.10 to 9.29, indicating high structural complexity. Premnaodoroside B exhibited the highest synthetic accessibility score of 9.29. This reflects the presence of multiple sugar moieties and complex ring systems. Pharmacokinetic radar profiles indicated that these compounds exceed optimal ranges in polarity and molecular size. High polarity limits passive diffusion across lipid membranes [67]. The presence of multiple glycosidic linkages increases structural flexibility and hydrogen bonding potential. These properties reduce oral bioavailability and systemic absorption [68]. The abundance of hydroxyl groups contributes to strong intermolecular interactions. These interactions may enhance binding affinity to certain biological targets. Structural rigidity in some iridoid cores may also influence binding orientation [69]. The balance between rigidity and flexibility affects interaction dynamics. Catalpol derivatives conjugated with aromatic acyl groups remain highly polar despite the presence of hydrophobic substituents. Their large molecular framework places them outside the preferred physicochemical window for small-molecule drug candidates. The very high synthetic accessibility values indicate that laboratory preparation or structural simplification would be challenging [70,71]. The data indicate that iridoid glycosides require optimization to improve drug-likeness and pharmacokinetic performance.

### 4.3. Phenylpropanoid Glycosides

Phenylpropanoid glycosides identified from *Premna serratifolia* include acteoside, forsythoside A, forsythoside B, isoacteoside, scroside E, campneoside I, and lavandulifolioside. These compounds exhibited molecular weights ranging from 624.6 to 756.7 g/mol. Most phenylpropanoid glycosides showed three Lipinski violations related to molecular weight, hydrogen bond donors, and hydrogen bond acceptors. These violations indicate high polarity and reduced membrane permeability. Lead-likeness analysis also revealed violations related to molecular size and rotatable bonds. These compounds exceeded the acceptable number of rotatable bonds above seven. Synthetic accessibility scores ranged from 6.37 to 7.28, indicating high structural complexity. Pharmacokinetic radar profiles indicated that these compounds fall outside optimal ranges for size and polarity. High polarity limits their diffusion across biological membranes. The presence of multiple hydroxyl groups increases hydrogen bonding interactions. These features support strong antioxidant and bioactive potential. High polarity also increases solubility in aqueous environments [72]. The aromatic phenylpropanoid core contributes to resonance stabilization. This stabilization may enhance radical scavenging ability. The glycosidic moiety influences solubility and transport behavior. Structural diversity among these compounds affects interaction specificity [73,74]. Caffeoyl-containing members such as acteoside and forsythosides combine aromaticity with extensive hydroxylation, creating a highly functional but bulky scaffold. Their physicochemical profile favors external or target-localized interactions more than passive oral absorption. The relatively high synthetic accessibility scores show that these molecules are structurally intricate and difficult to simplify without altering key functional groups [75]. The data indicate that phenylpropanoid glycosides require optimization to improve pharmacokinetic properties.

### 4.4. Terpenoids and Steroidal Compounds

Terpenoids identified from *Premna serratifolia* include 6-hydroxysalvinolone, citronellol, clerodin, clerodendrin A, nobilin D, and phytol. These compounds exhibited molecular weights ranging from 156.26 to 606.7 g/mol. Several terpenoids, such as 6-hydroxysalvinolone and nobilin D, satisfied Lipinski’s rule without violations. Citronellol showed lead-likeness violations related to low molecular weight and lipophilicity. Phytol exhibited violations related to high lipophilicity and excessive rotatable bonds. Clerodin and clerodendrin A showed limitations associated with molecular size and flexibility. Synthetic accessibility scores ranged from 2.61 to 7.47, depending on structural complexity. Diterpenoids generally showed higher scores compared to monoterpenes. Steroidal compounds such as sarsasapogenin exhibited violations related to high lipophilicity with MLOGP above 4.15. High lipophilicity influences solubility and absorption properties. Pharmacokinetic radar profiles indicated deviations from optimal lipophilicity ranges. These properties support interaction with hydrophobic regions of biological targets. Lipophilic compounds may exhibit strong membrane affinity. Excessive lipophilicity may reduce aqueous solubility [76]. Structural flexibility influences conformational adaptability. Rigid ring systems in steroids affect binding orientation [77]. Clerodane-type and related diterpenoids occupy an intermediate position between simple terpene alcohols and highly oxygenated large terpenoids. Nobilin D showed particularly favorable properties because it passed both Lipinski and lead-likeness filters with a low synthetic accessibility score of 2.94. By contrast, clerodendrin A combined high molecular weight with substantial oxygenation, producing multiple drug-likeness limitations and a synthetic accessibility score of 7.47. The data indicate that terpenoids and steroidal compounds require optimization to improve drug-likeness.

### 4.5. Phenolic Compounds and Small Molecules

Phenolic compounds identified from *Premna serratifolia* include caffeic acid, trans-cinnamic acid, and 4-hydroxy-3-methoxycinnamic acid. These compounds exhibited molecular weights ranging from 148.16 to 194.18 g/mol. All phenolic compounds satisfied Lipinski’s rule without violations. Lead-likeness analysis showed violations related to molecular weight below 250 g/mol. These values indicate that the compounds are small and structurally simple. Synthetic accessibility scores ranged from 1.67 to 1.93, indicating easy synthesis. Pharmacokinetic radar profiles indicated that these compounds fall within optimal ranges. Their small size supports high membrane permeability and absorption [78]. These compounds possess phenolic hydroxyl groups that contribute to antioxidant activity. Their simple structures allow rapid diffusion across biological membranes [79]. Aromatic rings contribute to electron delocalization and stability. These features enhance radical scavenging efficiency [80]. However, limited structural complexity may reduce target specificity. These compounds may act as supporting or synergistic agents. Structural modification may enhance their pharmacological activity. Functional group substitution can improve binding affinity [81]. Methoxy substitution in 4-hydroxy-3-methoxycinnamic acid slightly increases molecular size while maintaining favorable physicochemical behavior. Caffeic acid and trans-cinnamic acid showed the lowest synthetic accessibility scores in the dataset, reflecting highly tractable scaffolds for medicinal chemistry. Their simplicity makes them attractive starting points for derivatization aimed at improving potency without compromising permeability [82]. The data indicate that phenolic compounds provide a balance between simplicity, bioavailability, and modifiability.

## 5. Pharmacological Insights of Selected Bioactive Compounds

The selected bioactive compounds derived from *Premna serratifolia* and related phytochemicals were not only chosen based on reported biological activities but also through prior screening of physicochemical and drug-likeness parameters (Table 6 and Table 7). Compounds fulfilling favorable criteria such as Lipinski’s rule of five, lead-likeness compliance, moderate molecular weight, balanced lipophilicity, acceptable hydrogen bond donor and acceptor counts, and low to moderate synthetic accessibility were prioritized for further pharmacological interpretation. This selection strategy ensured that the discussed compounds possess both pharmacodynamic relevance and structural feasibility for drug development. The summarized studies consistently applied integrative methodologies combining network pharmacology with experimental validation. Network pharmacology served as a central framework to identify potential targets and uncover multi-target interactions across diverse disease contexts [83]. Molecular docking and pathway enrichment analyses further supported the mechanistic plausibility of compound–target interactions [84]. Experimental validation through in vitro and in vivo models strengthened the biological relevance of the predicted mechanisms [85]. Luteolin demonstrated anti-angiogenic activity through VEGFA inhibition, indicating its relevance in vascular-related diseases [86]. Acacetin exhibited broad anticancer potential by modulating EGFR, STAT3, and AKT1 signaling pathways [87]. Tricin showed significant tumor suppression through the regulation of PRKCA/SPHK/S1P signaling in lung cancer models [88]. Kaempferide enhanced osteogenic differentiation by activating the FoxO1/β-catenin signaling pathway and improving antioxidant defense [89]. Glycitein and daidzein displayed multitarget anticancer activity through the regulation of MAPK, PI3K-Akt, FoxO, and p53 signaling pathways [90,91]. These compounds collectively represent structurally favorable flavonoid scaffolds with experimentally validated biological effects. The evidence summarized highlights that these compounds were selected through a combination of drug-likeness filtering and mechanistic relevance. Such integration supports their potential as lead compounds rather than merely descriptive phytochemicals.

A deeper examination of their pharmacological profiles reveals a convergence of mechanisms across multiple disease models, particularly involving inflammation, oxidative stress, apoptosis, and dysregulated signaling pathways [95]. Despite differences in disease indications, many compounds target shared signaling axes, including PI3K-Akt, MAPK, FoxO, HIF-1α, ESR1-related pathways, and sphingolipid signaling [96]. Daidzein exemplifies this convergence by demonstrating both antioxidant and anticancer effects in glioblastoma through modulation of PI3K-Akt-associated targets [97]. Pectolinarigenin provides strong mechanistic evidence by directly interacting with HIF-1α, resulting in reduced oxidative stress and inflammation in nephrocalcinosis models [93]. Syringetin exhibits a distinct pharmacological profile by linking anticancer activity with analgesic effects through ESR1/PRDM2 signaling in bone cancer pain conditions [94]. Nobilin D contributes to the neuropharmacological aspect by targeting proteins associated with inflammation and neurotransmission in nervous system disorders [92]. These findings indicate that the selected compounds function as multitarget modulators rather than single-target inhibitors. Structural features such as moderate molecular size, absence of excessive glycosylation, and balanced polarity appear to support improved pharmacokinetic potential [98]. In contrast, highly polar or structurally complex compounds may exhibit biological activity but face limitations in membrane permeability and bioavailability [99]. The integration of computational prediction, docking validation, and biological assays increases confidence in the translational relevance of these compounds. The consistency between predicted targets and experimental outcomes further supports their mechanistic validity. These compounds also demonstrate versatility across therapeutic areas, including cancer, neurodegenerative disorders, renal injury, and bone-related diseases. This versatility reflects the adaptability of flavonoid-based scaffolds in modulating key biological pathways. The observed pharmacological effects may be partially attributed to the structural characteristics of flavonoids, isoflavonoids, and related phenolic metabolites present in *Premna serratifolia*. The presence of multiple hydroxyl groups enables antioxidant activity through hydrogen atom donation, electron transfer, and reactive oxygen species scavenging mechanisms, while aromatic ring systems facilitate interactions with protein targets through hydrophobic and π-related interactions. Methoxylated derivatives may further influence target affinity, membrane permeability, and metabolic stability. These structural features provide a plausible mechanistic basis for the diverse biological activities reported for the selected compounds across different experimental models. However, the proposed target interactions and signaling pathways should be interpreted with caution, as a substantial proportion of the available evidence is derived from computational analyses and indirect compound-level studies. Therefore, further experimental validation is required to confirm these mechanistic hypotheses.

## 6. Structural Feature Mapping of Selected Bioactive Compounds

**Figure 2:**
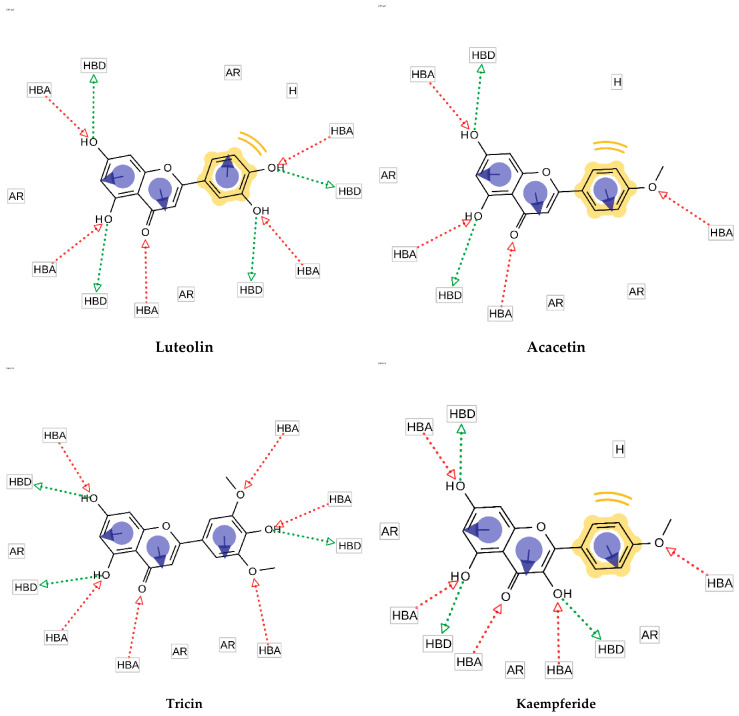

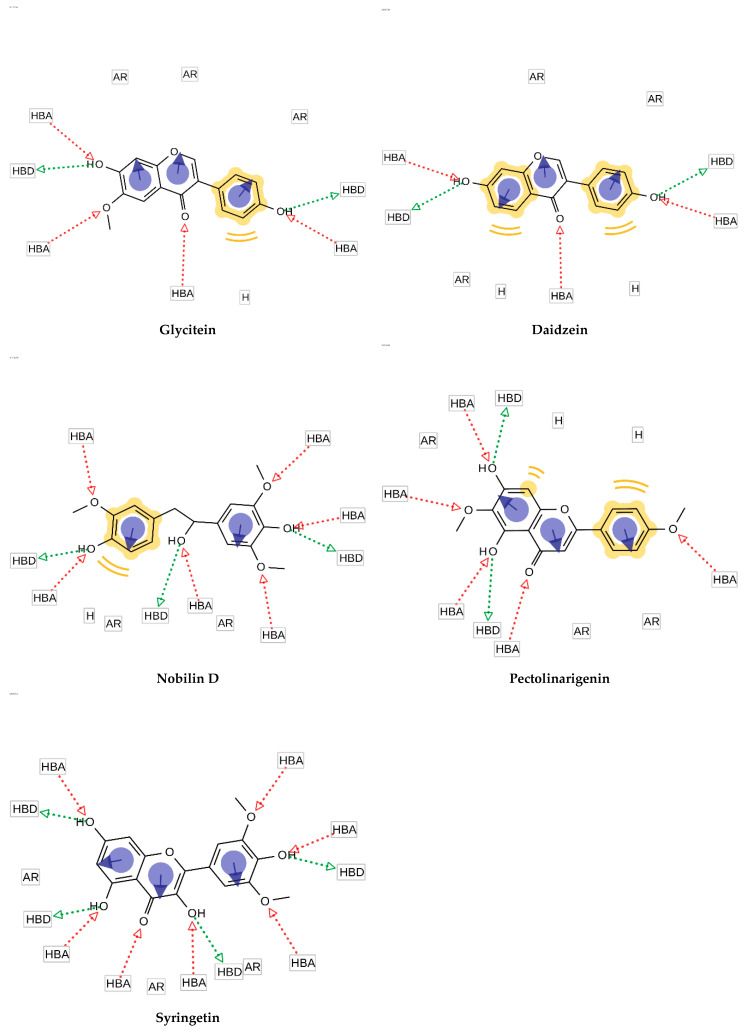
Two-Dimensional Structural Feature Annotation of Selected Bioactive Compounds Identified from *Premna serratifolia*, showing hydrogen bond acceptor (HBA), hydrogen bond donor (HBD), aromatic ring (AR), and hydrophobic (H) features. Red dashed arrows indicate HBA sites, green dashed arrows indicate HBD sites, blue circles represent aromatic ring centers, and yellow-highlighted regions denote hydrophobic aromatic moieties.

The two-dimensional structural feature annotation of selected bioactive compounds, highlighting key interaction features such as hydrogen bond donors, hydrogen bond acceptors, aromatic rings, and hydrophobic regions, which may contribute to their potential ligand-target interactions (Figure 2). The analyzed compounds include flavonoids, isoflavones, and related metabolites derived from *Premna serratifolia*. These structures contain molecular features that may contribute to ligand-target interactions and provide a qualitative basis for structural comparison among the selected compounds. The presence of multiple hydroxyl groups contributes significantly to hydrogen bonding capacity. These functional groups facilitate electrostatic and hydrogen bonding interactions with amino acid residues in protein targets. Aromatic rings observed in flavonoid structures enable π–π stacking interactions, enhancing binding stability [100,101]. Compounds such as tricin, kaempferide, glycitein, and daidzein display well-defined aromatic systems that support these interactions. Methoxylation patterns in certain compounds further influence electron distribution and interaction strength. The balance between hydrogen bonding features and aromaticity appears to determine pharmacological effectiveness [102]. Luteolin and acacetin exhibit multiple hydrogen bond donors and acceptors, indicating strong interaction potential. Glycitein and daidzein show slightly reduced polarity, which may improve membrane permeability. Kaempferide demonstrates a balanced combination of hydroxyl and methoxy groups, contributing to favorable interaction profiles. These structural variations indicate that minor substitutions can significantly influence binding behavior. The analyzed compounds exhibit similar aromatic and hydrogen-bonding features with variations in their functional group modifications. Glycitein and daidzein belong to the isoflavone class, whereas Nobilin D exhibits a structurally distinct framework compared with the flavonoid derivatives. The presence of these structural features suggests potential for molecular interactions; however, this alone does not demonstrate biological activity or specific target engagement.

A more detailed comparison shows that differences in the spatial distribution of pharmacophoric features influence interaction behavior across compound subclasses. Compounds with higher hydroxyl density exhibit stronger hydrogen bonding potential but also increased polarity [103]. This condition may enhance binding affinity while limiting passive membrane diffusion. Methoxylated compounds display improved lipophilicity, which supports better membrane penetration [104]. Nobilin D presents a distinct pharmacophore pattern characterized by increased hydrophobic character. This feature favors interaction with lipid-rich environments and hydrophobic binding sites. Pectolinarigenin combines hydrogen bonding capability with aromatic interactions, supporting its anti-inflammatory and antioxidant activities. Syringetin shows additional substitution patterns that enhance interaction versatility. The arrangement of hydrogen bond donors and acceptors influences target specificity and binding orientation. Aromatic rings contribute to the stabilization of ligand–protein complexes through π interactions [105]. Hydrophobic regions further strengthen binding within non-polar pockets [106]. However, these structural characteristics should be interpreted as indicators of potential interaction behavior rather than direct evidence of multitarget pharmacological activity. Experimental validation remains necessary to establish the biological relevance of these features. Structural balance between polarity and lipophilicity remains critical for optimal biological activity. Compounds with well-distributed pharmacophoric elements tend to exhibit more favorable interaction profiles [107]. These findings support the potential of selected *Premna serratifolia* compounds as lead structures for further drug development.

## 7. Future Perspectives and Discussion

**Figure 3:**
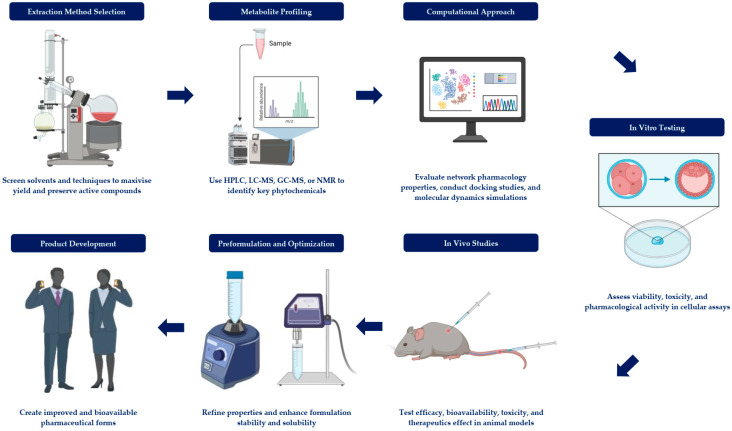
Proposed Workflow for The Discovery and Development of *Premna serratifolia*-Derived Bioactive Compounds, encompassing extraction, metabolite profiling, computational analyses (ADMET, network pharmacology, molecular docking, and molecular dynamics), in vitro and in vivo validation, preformulation optimization, and pharmaceutical development.

The development of bioactive compounds derived from *Premna serratifolia* requires a systematic and integrated workflow that combines experimental and computational approaches. Figure 3 illustrates a comprehensive pipeline encompassing extraction, metabolite profiling, computational analysis, biological validation, and formulation strategies. This workflow emphasizes the importance of selecting appropriate extraction methods to maximize yield while preserving bioactive constituents. The choice of solvent polarity and extraction technique directly influences the chemical composition of the extract [108]. Following extraction, metabolite profiling using advanced analytical techniques such as LC-MS, GC-MS, and NMR enables accurate identification of phytochemical constituents. This step is essential for establishing a chemical fingerprint and selecting promising candidate compounds [21]. Based on the reviewed evidence, leaf-derived extracts, particularly ethanol and ethyl acetate fractions, appear to be among the most promising sources of bioactive constituents, as reflected by their consistently reported antioxidant, antidiabetic, and other pharmacologically relevant activities. Flavonoids and isoflavonoids, including luteolin, acacetin, tricin, kaempferide, glycitein, and related metabolites, represent the most extensively characterized compound classes and may serve as priority candidates for future investigations.

Computational approaches, including network pharmacology, molecular docking, and molecular dynamics simulations, provide mechanistic insights into compound–target interactions. These methods enable identification of multitarget effects and key signaling pathways associated with disease modulation [109]. The integration of docking and molecular dynamics enhances the reliability of predicted binding interactions. Free-energy calculations such as MM-PBSA or MM-GBSA further improve compound prioritization [110]. In vitro testing plays a critical role in validating predicted biological activities, including cytotoxic, antioxidant, and anti-inflammatory effects. This stage also allows evaluation of dose-dependent responses and cellular mechanisms [111]. Future validation efforts should prioritize activities supported by experimental evidence, particularly antioxidant, antidiabetic, anti-inflammatory, and anticancer effects, followed by detailed mechanistic investigations and pharmacokinetic evaluations of isolated compounds. In vivo studies provide confirmation of therapeutic efficacy, pharmacokinetics, and safety under physiological conditions [112]. These experimental stages bridge computational predictions with biological outcomes. Preformulation and optimization processes are required to improve solubility, stability, and drug delivery performance [113].

The multitarget nature of *Premna serratifolia*-derived compounds provides a strategic advantage in addressing complex diseases involving multiple dysregulated pathways. Many compounds modulate central signaling networks such as PI3K-Akt, MAPK, FoxO, and HIF-1α pathways. These pathways regulate key biological processes including proliferation, apoptosis, oxidative stress, inflammation, and cellular survival. Such broad activity profiles support their application in cancer, neurodegenerative disorders, and metabolic diseases [114,115]. However, the complexity of multitarget interactions presents challenges in defining precise mechanisms of action. Systems biology approaches and integrative omics techniques such as transcriptomics and proteomics can help clarify these mechanisms [116]. Structural differences between aglycone and glycosylated compounds significantly influence pharmacokinetic behavior and biological activity. Aglycone compounds generally exhibit better membrane permeability and drug-likeness profiles [117]. Structure–activity relationship analysis is essential to identify functional groups responsible for biological effects. Structural optimization strategies may improve lipophilicity and reduce excessive polarity [118]. Artificial intelligence and machine learning approaches may accelerate compound optimization and target prediction [119]. Standardization of computational and experimental methodologies is necessary to improve reproducibility. Several limitations should also be considered when interpreting the current body of evidence. The reviewed studies employed heterogeneous extraction methods, solvents, plant materials, and biological assays, which make direct comparisons between studies challenging. Variability in activity units and reporting formats further complicates cross-study evaluation. In addition, many metabolite identifications were based on LC-MS annotation and database matching rather than confirmation using authentic reference standards. Most of the available biological evidence remains limited to in vitro investigations, while in vivo validation is comparatively scarce and clinical studies are currently unavailable. Furthermore, ADMET prediction, molecular docking, molecular dynamics simulations, and network pharmacology analyses provide valuable mechanistic hypotheses; however, these approaches remain predictive in nature and require further experimental confirmation. The identification of reliable biomarkers will support the evaluation of therapeutic efficacy. Interdisciplinary collaboration will further strengthen drug development efforts. These integrated strategies support the advancement of *Premna serratifolia* compounds as promising candidates for pharmaceutical development.

## 8. Conclusions

The compiled findings provide a comprehensive overview of the phytochemical characteristics, biological activities, structural properties, and pharmacological potential of bioactive compounds derived from *Premna serratifolia*. Variations in extraction methods and solvent polarity were shown to influence the yield and composition of phytochemicals, which in turn affected biological activity profiles. The distribution of phenolic and flavonoid compounds was closely associated with antioxidant, antimicrobial, and enzyme inhibitory activities. Chemical profiling revealed a diverse group of metabolites, including flavonoids, iridoid glycosides, phenylpropanoid glycosides, terpenoids, and simple phenolic compounds. Structural classification indicated that flavonoid aglycones generally possess more favorable physicochemical characteristics than glycosylated derivatives. Evaluation based on drug-likeness parameters, including Lipinski’s rule of five, lead-likeness, molecular weight, lipophilicity, hydrogen bonding capacity, and synthetic accessibility, supported the prioritization of selected compounds. These criteria enabled the identification of molecules with balanced structural and pharmacokinetic properties. Available evidence suggests that several compounds reported from *Premna serratifolia* may interact with multiple biological targets and signaling pathways; however, a substantial proportion of the evidence is derived from computational analyses or studies conducted on isolated compounds outside the direct context of *Premna serratifolia*. Accordingly, many of the reported target-specific mechanisms should be regarded as indirect compound-level evidence rather than direct experimental evidence obtained from *Premna serratifolia*. The pharmacological evidence demonstrated that the selected compounds act through multitarget mechanisms across various disease models. Key signaling pathways involved include PI3K-Akt, MAPK, FoxO, HIF-1α, and ESR1-associated networks. These pathways regulate critical biological processes such as proliferation, apoptosis, oxidative stress, inflammation, and cellular survival. Structural features were found to play a significant role in determining interaction efficiency and biological activity. Aglycone flavonoids consistently exhibited improved membrane permeability and interaction profiles compared to more complex molecules. The integration of phytochemical analysis, computational modeling, and biological validation enhanced the reliability of compound selection. This combined approach provides a structured framework for natural product-based drug discovery. Further optimization through structural modification, advanced computational techniques, and formulation development is necessary to improve therapeutic performance. Direct evidence from *Premna serratifolia* extracts supports antioxidant, antimicrobial, and enzyme inhibitory activities, whereas many target-specific mechanisms and disease-related effects are derived from indirect compound-level evidence obtained from studies of individual metabolites in other biological systems. Several limitations should also be acknowledged, including heterogeneity in extraction methodologies, lack of extract standardization, variability in biological assay conditions and reporting units, tentative metabolite identification based on LC–MS annotation, limited in vivo validation, and the absence of clinical studies. In addition, many biological activities attributed to individual constituents are inferred from studies conducted in different botanical or experimental contexts, which should be interpreted with caution. Furthermore, computational approaches, including ADMET prediction, molecular docking, molecular dynamics simulations, and network pharmacology analyses, provide valuable mechanistic insights and prioritization strategies but remain predictive in nature. Therefore, these findings require rigorous experimental validation through in vitro, in vivo, pharmacokinetic, toxicity, and ultimately clinical studies before definitive therapeutic applications can be established. Therefore, while the available evidence highlights the considerable potential of *Premna serratifolia* as a valuable source of bioactive compounds, the current conclusions should be considered preliminary, and further pharmacokinetic, toxicity, in vivo, and clinical investigations are required before definitive pharmaceutical applications can be established.

## Figures and Tables

**Table 3:** Identified Metabolites in the Fraction and Extract of *Premna serratifolia* Leaves Based on LC–MS and UPLC-Q-Orbitrap HRMS Analysis.

**No**	**Compound Name**	**Analytical Method**	**Sample/Fraction**	**Retention Time (min)**	**Theoretical *m*/*z***	**Experimental *m*/*z***	**Accuracy (ppm)**	**Major MS/MS Fragments (*m*/*z*)**	**Identification Confidence**	**Reference**
1	Centaureidin	UHPLC-Q-Orbitrap HRMS	Ethyl acetate fraction of *Premna serratifolia* leaves	14.54	360.08452	360.08306	−4.05	346.06784; 209.63461	Tentative (Database Match)	[47]
2	Chrysin			11.15, 15.12	254.05791	254.05653	−5.43	209.05879; 153.01813	Level 2 (MS/MS + Literature Match)	
3	Glycitein			15.27, 8.35, 15.31, 15.27	284.06847	284.06674	−6.11	270.05167; 242.05692	Level 2 (MS/MS + Literature Match)	
4	3,5,4′-Trimethoxy-6,7methylenedioxyflavone			13.9	356.0896	356.08853	−3.02	339.08603; 311.09122; 297.07544	Putative Novel Compound (Level 3)	
5	Kaempferide			15.63	300.06339	300.0624	−3.3	151.03915; 107.04931	Tentative (Database Match)	
6	Pectolinarigenin			16.1	314.07904	314.07966	1.97	298.04794; 283.02451	Level 2 (MS/MS + Literature Match)	
7	Syringetin			12.34, 12.29	346.06887	346.06707	−5.2	301.06970; 151.03864	Level 2 (MS/MS + Literature Match)	
8	Tricin			12.71	330.07395	330.07257	−4.2	316.05722; 98.98434	Level 2 (MS/MS + Literature Match)	
9	Casticin			15.01, 14.99	374.10017	374.0989	−3.39	360.08289; 342.07266	Level 2 (MS/MS + Literature Match)	
**No**	**Compound Name**	**Analytical Method**	**Sample/Fraction**	**Rt (min)**	**Ion Mode**	**Theoretical *m*/*z***	**Experimental *m*/*z***	**Mass Error (ppm)**	**Confidence Level**	**Reference**
10	Diosmin	LC-MS	Stem ethyl acetate extract (S-EA) fraction of *Premna serratifolia*	14.428	+ve	608.1741	608.1741	−0.02	Level 2 (MS/MS + Literature Match)	[58]
11	Isorhamnetin-3-O-glucoside			13.684	−ve	478.1111	478.11	−2.29	Level 2 (MS/MS + Literature Match)	
12	Kaempferol 3,7-di-O-alpha-L-rhamnoside			12.081	+ve	578.1636	578.1641	0.9	Level 2 (MS/MS + Literature Match)	
13	Luteolin			18.273	+ve	286.0477	286.0467	−3.63	Level 2 (MS/MS + Literature Match)	
14	10-O-trans-p-coumaroyl-catalpol			3.446	−ve	508.1581	508.1582	0.19	Level 3 (Tentative Candidate)	
15	10-O-trans-p-methoxycinamoyl-catalpol			14.844	+ve	522.1737	522.1725	−2.26	Level 3 (Tentative Candidate)	
16	4-hydroxy-E-globularinin			11.764	−ve	526.1686	526.1669	−3.38	Level 3 (Tentative Candidate)	
17	6-O-(3″-O-trans-p-coumaroyl)-alpha-Lrhamnopyranosyl catalpol			13.279	+ve	654.216	654.2156	−0.52	Level 3 (Tentative Candidate)	
18	Premnaodoroside B			20.476	−ve	890.4147	890.4138	−1.02	Level 3 (Tentative Candidate)	
19	Premnazole			2.476	+ve	141.0426	141.0421	−3.33	Level 3 (Tentative Candidate)	
20	Caffeic acid			2.326	+ve	180.0423	180.0423	0.46	Level 3 (Tentative Candidate)	
21	Acteoside			14.133	−ve	624.2054	624.2058	0.6	Level 3 (Tentative Candidate)	
22	6-hydroxysalvinolone			24.97	−ve	330.1831	330.1827	−1.18	Level 3 (Tentative Candidate)	
23	Citronellol			12.847	+ve	154.1358	154.1354	−2.64	Level 3 (Tentative Candidate)	
24	Clerodin			20.587	+ve	434.2305	434.2298	−1.46	Level 3 (Tentative Candidate)	
25	Clerodendrin A			19.31	−ve	606.2676	606.2685	1.43	Level 3 (Tentative Candidate)	
**No**	**Compound Name**	**Analytical Method**	**Sample/Fraction**	**RT (min)**	**Ion Mode**	**Observed *m*/*z***	**Major Fragments (*m*/*z*)**		**Confidence Level**	**Reference**
26	Scroside E	LC-QTOF-MS/MS	Ethanol extract of *Premna serratifolia* leaves	7.22	+	673	672, 293, 147		Level 2 (MS/MS + Literature Match)	[1]
27	Campneoside I			8.17	−	653	653		Level 2 (MS/MS + Literature Match)	
28	Forsythoside B			8.32	−	756	755		Level 2 (MS/MS + Literature Match)	
29	Forsythoside A			8.49	+	625	663, 642, 608, 325, 163		Level 2 (MS/MS + Literature Match)	
30	Isoacteoside			8.5	−	623	623		Level 2 (MS/MS + Literature Match)	
31	Lavandulifolioside			8.6	+	757	774, 325		Level 2 (MS/MS + Literature Match)	
32	Nobilin D			12.24	+	321	307, 161		Level 2 (MS/MS + Literature Match)	
**No**	**Compound Name**	**Analytical Method**	**Sample/Fraction**	**RT (min)**			**Ion Mode**		**Confidence Level**	
33	4-Hydroxy-3-methoxycinnamic acid	UPLC/MS–MS	Aqueous root extract of *Premna serratifolia*	8.68			+ve		Level 3 (Tentative Candidate)	[16]
34	Linarin			9.33			+ve		Level 3 (Tentative Candidate)	
35	Peonidin-3,5-O-di-beta-glucopyranoside			10.2			+ve		Level 3 (Tentative Candidate)	
36	trans-Cinnamic acid			13.1			+ve		Level 3 (Tentative Candidate)	
37	Daidzein			13.1			−ve		Level 3 (Tentative Candidate)	
38	Saponarin			13.6			−ve		Level 3 (Tentative Candidate)	
39	Homoorietin			14			−ve		Level 3 (Tentative Candidate)	
40	Acacetin			14.4			−ve		Level 3 (Tentative Candidate)	
41	Sarsasapogenin			14.9			−ve		Level 3 (Tentative Candidate)	
42	Phytol			15.1			−ve		Level 3 (Tentative Candidate)	
43	Sissotrin			16.2			−ve		Level 3 (Tentative Candidate)	

**Table 4:** Chemical Classification and Structural Characteristics of Major Flavonoids Identified from *Premna serratifolia*.

No	Compound Name	Analytical Method	Sample/Fraction	Compound Class	Subclass	Molecular Formula	Molecular Weight	SMILES	Reference
1	Centaureidin	UHPLC-Q-Orbitrap HRMS	Ethyl acetate fraction of *Premna serratifolia* leaves	Flavonoid	Flavone (polymethoxylated)	C_18_H_16_O_8_	360.3 g/mol	COC1=C(C=C(C=C1)C2=C(C(=O)C3=C(O2)C=C(C(=C3O)OC)O)OC)O	[47]
2	Chrysin			Flavonoid	Flavone	C_15_H_10_O_4_	254.24 g/mol	C1=CC=C(C=C1)C2=CC(=O)C3=C(C=C(C=C3O2)O)O	
3	Glycitein			Flavonoid	Isoflavone glycoside	C_16_H_12_O_5_	284.26 g/mol	COC1=C(C=C2C(=C1)C(=O)C(=CO2)C3=CC=C(C=C3)O)O	
4	3,5,4′-Trimethoxy-6,7methylenedioxyflavone			Flavonoid	Flavone	C_19_H_16_O_7_	356.33 g/mol	COC1=CC=C(C=C1)C1=C(OC)C(=O)C2=C(OC)C3=C(OCO3)C=C2O1	
5	Kaempferide			Flavonoid	Flavonol	C_16_H_12_O_6_	300.26 g/mol	COC1=CC=C(C=C1)C2=C(C(=O)C3=C(C=C(C=C3O2)O)O)O	
6	Pectolinarigenin			Flavonoid	Flavone	C_17_H_14_O_6_	314.29 g/mol	COC1=CC=C(C=C1)C2=CC(=O)C3=C(O2)C=C(C(=C3O)OC)O	
7	Syringetin			Flavonoid	Flavonol	C_17_H_14_O_8_	346.3 g/mol	COC1=CC(=CC(=C1O)OC)C2=C(C(=O)C3=C(C=C(C=C3O2)O)O)O	
8	Tricin			Flavonoid	Flavone	C_17_H_14_O_7_	330.29 g/mol	COC1=CC(=CC(=C1O)OC)C2=CC(=O)C3=C(C=C(C=C3O2)O)O	
9	Casticin			Flavonoid	Flavone (polymethoxylated)	C_19_H_18_O_8_	374.3 g/mol	COC1=C(C=C(C=C1)C2=C(C(=O)C3=C(C(=C(C=C3O2)OC)OC)O)OC)O	
10	Diosmin	LC-MS	Stem ethyl acetate extract (S-EA) fraction of *Premna serratifolia*	Flavonoid	Flavone glycoside	C_28_H_32_O_15_	608.5 g/mol	C[C@H]1[C@@H]([C@H]([C@H]([C@@H](O1)OC[C@@H]2[C@H]([C@@H]([C@H]([C@@H](O2)OC3=CC(=C4C(=C3)OC(=CC4=O)C5=CC(=C(C=C5)OC)O)O)O)O)O)O)O)O	[58]
11	Isorhamnetin-3-O-glucoside			Flavonoid	Flavonol glycoside	C_22_H_22_O_12_	478.4 g/mol	COC1=C(C=CC(=C1)C2=C(C(=O)C3=C(C=C(C=C3O2)O)O)O[C@H]4[C@@H]([C@H]([C@@H]([C@H](O4)CO)O)O)O)O	
12	Kaempferol 3,7-di-O-alpha-L-rhamnoside			Flavonoid	Flavonol glycoside	C_27_H_30_O_14_	578.5 g/mol	C[C@H]1[C@@H]([C@H]([C@H]([C@@H](O1)OC2=CC(=C3C(=C2)OC(=C(C3=O)O[C@H]4[C@@H]([C@@H]([C@H]([C@@H](O4)C)O)O)O)C5=CC=C(C=C5)O)O)O)O)O	
13	Luteolin			Flavonoid	Flavone	C_15_H_10_O_6_	286.24 g/mol	C1=CC(=C(C=C1C2=CC(=O)C3=C(C=C(C=C3O2)O)O)O)O	
14	10-O-trans-p-coumaroyl-catalpol			Iridoid glycoside	Phenylpropanoid-conjugated iridoid	C_24_H_28_O_12_	508.47 g/mol	OC[C@H]1O[C@@H](O[C@@H]2OC=C[C@H]3[C@H](O)[C@@H]4O[C@]4(COC(=O)\C=C\C4=CC=C(O)C=C4)[C@@H]23)[C@H](O)[C@@H](O)[C@@H]1O	
15	10-O-trans-p-methoxycinamoyl-catalpol			Iridoid glycoside	Phenylpropanoid-conjugated iridoid	C_25_H_30_O_12_	522.5 g/mol	COC1=CC=C(C=C1)/C=C/C(=O)OC[C@@]23[C@@H]4[C@@H](C=CO[C@H]4O[C@H]5[C@@H]([C@H]([C@@H]([C@H](O5)CO)O)O)O)[C@@H]([C@@H]2O3)O	
16	4-hydroxy-E-globularinin			Lignan/Phenylpropanoid	Lignan derivative	C_24_H_30_O_13_	526.49 g/mol	C(OC(/C=C/C1=CC=C(O)C=C1)=O)[C@]2(O)[C@@]3([C@]([C@H](O)[C@@H]2O)(C=CO[C@H]3O[C@@H]4O[C@H](CO)[C@@H](O)[C@H](O)[C@H]4O)[H])[H]	
17	6-O-(3″-O-trans-p-coumaroyl)-alpha-Lrhamnopyranosyl catalpol			Iridoid glycoside	Phenylpropanoid-conjugated iridoid	C_39_H_44_O_17_	784.76 g/mol	C[C@@H]1O[C@@H](O[C@@H]2[C@@H]3O[C@]3(CO)[C@@H]3[C@H]2C=CO[C@H]3O[C@@H]2O[C@H](COC(=O)/C=C/c3ccccc3)[C@@H](OC(=O)/C=C/c3ccc(O)cc3)[C@H](O)[C@H]2O)[C@H](O)[C@H](O)[C@H]1O	
18	Premnaodoroside B			Iridoid glycoside	Iridoid derivative	C_42_H_66_O_20_	890.96 g/mol	CC(CCCC(C)COC(=O)C1=CO[C@@H](O[C@@H]2O[C@H](CO)[C@@H](O)[C@H](O)[C@H]2O)[C@@H]2[C@H](C)[C@@H](O)C[C@H]12)CCOC(=O)C1=CO[C@@H](O[C@@H]2O[C@H](CO)[C@@H](O)[C@H](O)[C@H]2O)[C@H]2[C@@H]1CC[C@]2(C)O	
19	Premnazole			Alkaloid	Heterocyclic alkaloid	C_6_H_7_NO_3_	145.15 g/mol	[2H]C1=C(ON=C1C(=O)OC)C([2H])([2H])[2H]	
20	Caffeic acid			Phenolic compound	Hydroxycinnamic acid	C_9_H_8_O_4_	180.16 g/mol	C1=CC(=C(C=C1/C=C/C(=O)O)O)O	
21	Acteoside			Phenylpropanoid glycoside	Caffeoyl phenylethanoid glycoside	C_29_H_36_O_15_	624.6 g/mol	C[C@H]1[C@@H]([C@H]([C@H]([C@@H](O1)O[C@@H]2[C@H]([C@@H](O[C@@H]([C@H]2OC(=O)/C=C/C3=CC(=C(C=C3)O)O)CO)OCCC4=CC(=C(C=C4)O)O)O)O)O)O	
22	6-hydroxysalvinolone			Terpenoid	Diterpenoid	C_20_H_28_O_3_	316.4 g/mol	CC(C)C1=C(C(=C2C(=C1)CC(=C3[C@@]2(CCCC3(C)C)C)O)O)O	
23	Citronellol			Terpenoid	Monoterpene alcohol	C_10_H_20_O	156.26 g/mol	CC(CCC=C(C)C)CCO	
24	Clerodin			Terpenoid	Diterpenoid (clerodane type)	C_24_H_34_O_7_	434.5 g/mol	C[C@@H]1C[C@@H]([C@@]2([C@@H]([C@@]1(C)[C@@H]3C[C@H]4C=CO[C@H]4O3)CCC[C@]25CO5)COC(=O)C)OC(=O)C	
25	Clerodendrin A			Terpenoid	Diterpenoid	C_31_H_42_O_12_	606.7 g/mol	CC[C@](C)(C(=O)O[C@H]1[C@@H](C[C@@H]2[C@@](C(=C[C@@H]([C@]2([C@@]13CO3)COC(=O)C)OC(=O)C)C)(C)[C@@H]4C[C@H]5C=CO[C@H]5O4)O)OC(=O)C	
26	Scroside E	LC-QTOF-MS/MS	Ethanol extract of *Premna serratifolia* leaves	Phenylpropanoid glycoside	Phenylethanoid glycoside	C_30_H_38_O_17_	670.61 g/mol	OCCc1ccc(O)c(O)c1O[C@H]1O[C@@H](COC(=O)/C=C/c2cc(OC)c(O)cc2)[C@H](O[C@@H]2O[C@H](CO)[C@@H](O)[C@H](O)[C@H]2O)[C@@H](O)[C@H]1O	[1]
27	Campneoside I			Phenylpropanoid glycoside	Phenylethanoid glycoside	C_30_H_38_O_16_	654.6 g/mol	C[C@H]1[C@@H]([C@H]([C@H]([C@@H](O1)O[C@@H]2[C@H]([C@@H](O[C@@H]([C@H]2OC(=O)/C=C/C3=CC(=C(C=C3)O)O)CO)OCC(C4=CC(=C(C=C4)O)O)OC)O)O)O)O	
28	Forsythoside B			Phenylpropanoid glycoside	Caffeoyl glycoside	C_34_H_44_O_19_	756.7 g/mol	C[C@H]1[C@@H]([C@H]([C@H]([C@@H](O1)O[C@@H]2[C@H]([C@@H](O[C@@H]([C@H]2OC(=O)/C=C/C3=CC(=C(C=C3)O)O)CO[C@H]4[C@@H]([C@](CO4)(CO)O)O)OCCC5=CC(=C(C=C5)O)O)O)O)O)O	
29	Forsythoside A			Phenylpropanoid glycoside	Caffeoyl glycoside	C_29_H_36_O_15_	624.6 g/mol	C[C@H]1[C@@H]([C@H]([C@H]([C@@H](O1)OC[C@@H]2[C@H]([C@@H]([C@H]([C@@H](O2)OCCC3=CC(=C(C=C3)O)O)O)O)OC(=O)/C=C/C4=CC(=C(C=C4)O)O)O)O)O	
30	Isoacteoside			Phenylpropanoid glycoside	Acteoside isomer	C_29_H_36_O_15_	624.6 g/mol	C[C@H]1[C@@H]([C@H]([C@H]([C@@H](O1)O[C@H]2[C@@H]([C@H](O[C@H]([C@@H]2O)OCCC3=CC(=C(C=C3)O)O)COC(=O)/C=C/C4=CC(=C(C=C4)O)O)O)O)O)O	
31	Lavandulifolioside			Phenylpropanoid glycoside	Phenylethanoid glycoside	C_34_H_44_O_19_	756.7 g/mol	C[C@H]1[C@@H]([C@H]([C@H]([C@@H](O1)O[C@@H]2[C@H]([C@@H](O[C@@H]([C@H]2OC(=O)/C=C/C3=CC(=C(C=C3)O)O)CO)OCCC4=CC(=C(C=C4)O)O)O)O[C@H]5[C@H]([C@@H]([C@@H](CO5)O)O)O)O)O	
32	Nobilin D			Terpenoid	Diterpenoid	C_17_H_20_O_6_	320.3 g/mol	COC1=CC(=CC(=C1O)OC)C(CC2=CC(=C(C=C2)O)OC)O	
33	4-Hydroxy-3-methoxycinnamic acid	UPLC/MS–MS	Aqueous root extract of *Premna serratifolia*	Phenolic compound	Hydroxycinnamic acid (Ferulic acid type)	C_10_H_10_O_4_	194.18 g/mol	COC1=C(C=CC(=C1)/C=C/C(=O)O)O	[16]
34	Linarin			Flavonoid	Flavone glycoside	C_28_H_32_O_14_	592.5 g/mol	C[C@H]1[C@@H]([C@H]([C@H]([C@@H](O1)OC[C@@H]2[C@H]([C@@H]([C@H]([C@@H](O2)OC3=CC(=C4C(=C3)OC(=CC4=O)C5=CC=C(C=C5)OC)O)O)O)O)O)O)O	
35	Peonidin-3,5-O-di-beta-glucopyranoside			Flavonoid	Anthocyanin glycoside	C_28_H_33_O_16_^+^	625.6 g/mol	COC1=C(C=CC(=C1)C2=C(C=C3C(=CC(=CC3=[O+]2)O)O[C@H]4[C@@H]([C@H]([C@@H]([C@H](O4)CO)O)O)O)O[C@H]5[C@@H]([C@H]([C@@H]([C@H](O5)CO)O)O)O)O	
36	trans-Cinnamic acid			Phenolic compound	Phenylpropanoid acid	C_9_H_8_O_2_	148.16 g/mol	C1=CC=C(C=C1)/C=C/C(=O)O	
37	Daidzein			Flavonoid	Isoflavone	C_15_H_10_O_4_	254.24 g/mol	C1=CC(=CC=C1C2=COC3=C(C2=O)C=CC(=C3)O)O	
38	Saponarin			Flavonoid	Flavone glycoside	C_27_H_30_O_15_	594.5 g/mol	C1=CC(=CC=C1C2=CC(=O)C3=C(C(=C(C=C3O2)O[C@H]4[C@@H]([C@H]([C@@H]([C@H](O4)CO)O)O)O)[C@H]5[C@@H]([C@H]([C@@H]([C@H](O5)CO)O)O)O)O)O	
39	Homoorietin			Flavonoid	C-glycosyl flavone	C_21_H_20_O_11_	448.4 g/mol	C1=CC(=C(C=C1C2=CC(=O)C3=C(O2)C(=C(C=C3O)O)C4C(C(C(C(O4)CO)O)O)O)O)O	
40	Acacetin			Flavonoid	Flavone	C_16_H_12_O_5_	284.26 g/mol	COC1=CC=C(C=C1)C2=CC(=O)C3=C(C=C(C=C3O2)O)O	
41	Sarsasapogenin			Steroidal compound	Steroidal sapogenin	C_27_H_44_O_3_	416.6 g/mol	C[C@H]1CC[C@@]2([C@H]([C@H]3[C@@H](O2)C[C@@H]4[C@@]3(CC[C@H]5[C@H]4CC[C@H]6[C@@]5(CC[C@@H](C6)O)C)C)C)OC1	
42	Phytol			Terpenoid	Diterpene alcohol	C_20_H_40_O	296.5 g/mol	C[C@@H](CCC[C@@H](C)CCC/C(=C/CO)/C)CCCC(C)C	
43	Sissotrin			Flavonoid	Isoflavone glycoside	C_22_H_22_O_10_	446.4 g/mol	COC1=CC=C(C=C1)C2=COC3=CC(=CC(=C3C2=O)O)O[C@H]4[C@@H]([C@H]([C@@H]([C@H](O4)CO)O)O)O	

**Table 5:** ADMET-Related Drug-Likeness and Pharmacokinetic Profiling of Selected *Premna serratifolia* Metabolites.

No	Compound Name	Molecular Structure (2D)	Pharmacokinetic Radar Profile	Lipinski Rule	Lead-Likeness	Synthetic Accessibility Score
1	Centaureidin	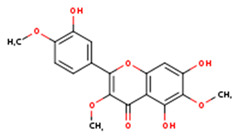	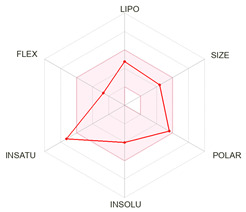	Yes; 0 violation	No; 1 violation: MW > 350	3.57
2	Chrysin	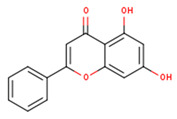	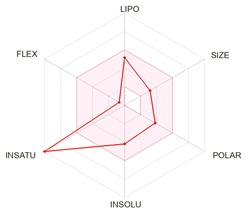	Yes; 0 violation	No; 1 violation: XLOGP3 > 3.5	2.93
3	Glycitein	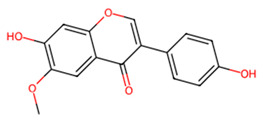	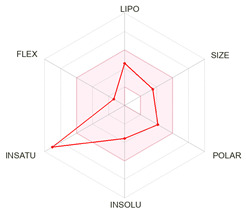	Yes; 0 violation	Yes	2.95
4	3,5,4′-Trimethoxy-6,7methylenedioxyflavone	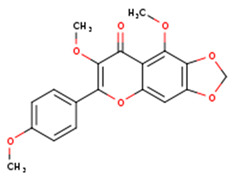	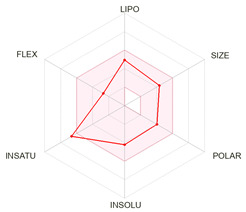	Yes; 0 violation	No; 1 violation: MW > 350	3.67
5	Kaempferide	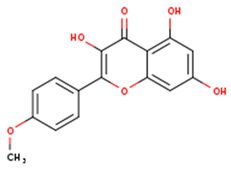	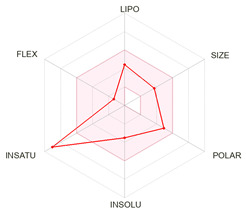	Yes; 0 violation	Yes	3.16
6	Pectolinarigenin	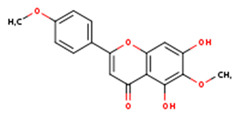	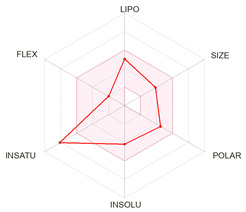	Yes; 0 violation	Yes	3.22
7	Syringetin	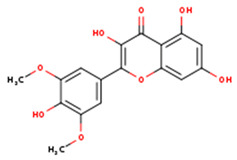	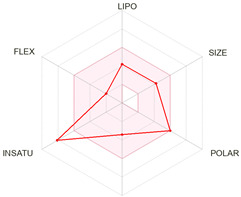	Yes; 0 violation	Yes	3.41
8	Tricin	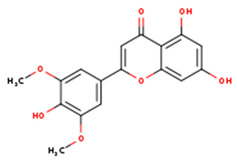	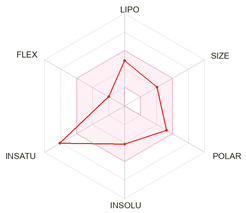	Yes; 0 violation	Yes	3.21
9	Casticin	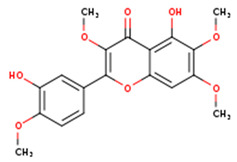	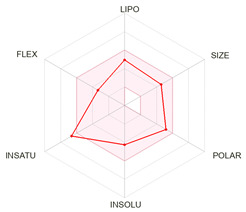	Yes; 0 violation	No; 1 violation: MW > 350	3.71
10	Diosmin	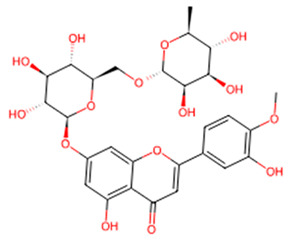	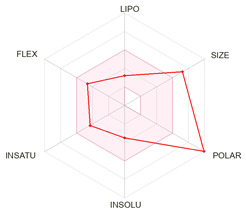	No; 3 violations: MW > 500, NorO > 10, NHorOH > 5	No; 1 violation: MW > 350	6.48
11	Isorhamnetin-3-O-glucoside	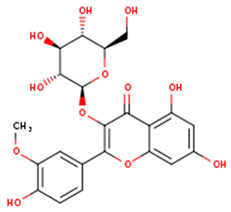	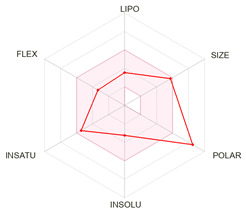	No; 2 violations: NorO > 10, NHorOH > 5	No; 1 violation: MW > 350	5.44
12	Kaempferol 3,7-di-O-alpha-L-rhamnoside	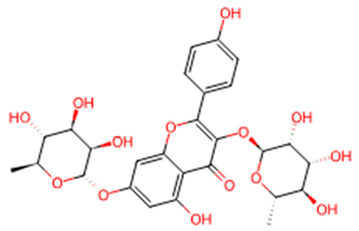	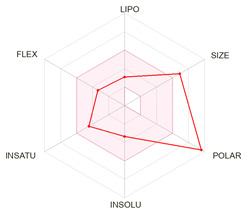	No; 3 violations: MW > 500, NorO > 10, NHorOH > 5	No; 1 violation: MW > 350	6.48
13	Luteolin	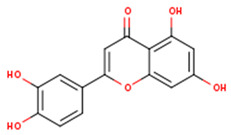	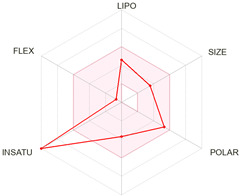	Yes; 0 violation	Yes	3.02
14	10-O-trans-p-coumaroyl-catalpol	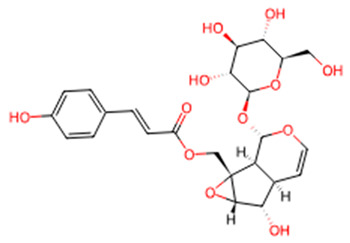	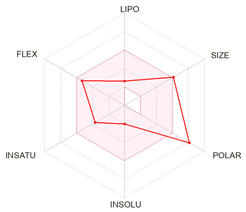	No; 3 violations: MW > 500, NorO > 10, NHorOH > 5	No; 2 violations: MW > 350, Rotors > 7	6.10
15	10-O-trans-p-methoxycinamoyl-catalpol	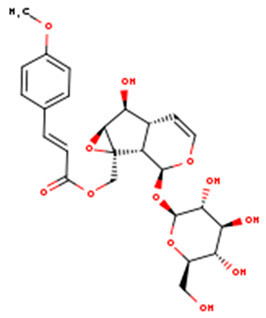	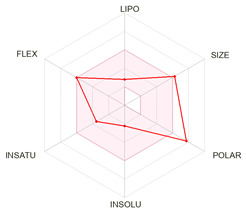	No; 2 violations: MW > 500, NorO > 10	No; 2 violations: MW > 350, Rotors > 7	6.19
16	4-hydroxy-E-globularinin	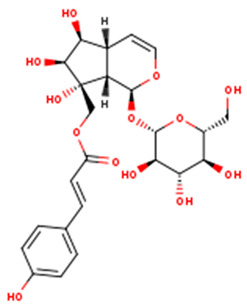	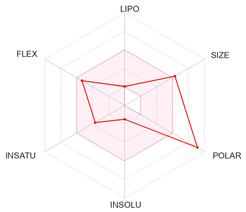	No; 3 violations: MW > 500, NorO > 10, NHorOH > 5	No; 2 violations: MW > 350, Rotors > 7	6.24
17	6-O-(3″-O-trans-p-coumaroyl)-alpha-Lrhamnopyranosyl catalpol	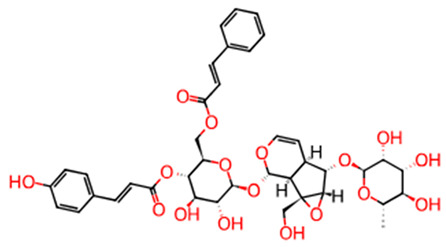	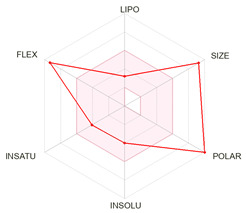	No; 3 violations: MW > 500, NorO > 10, NHorOH > 5	No; 2 violations: MW > 350, Rotors > 7	7.85
18	Premnaodoroside B	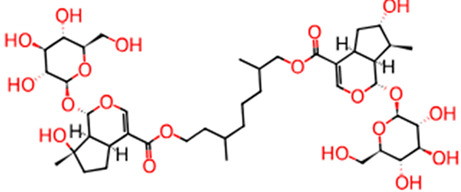	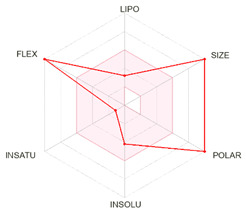	No; 3 violations: MW > 500, NorO > 10, NHorOH > 5	No; 2 violations: MW > 350, Rotors > 7	9.29
19	Premnazole	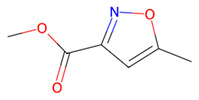	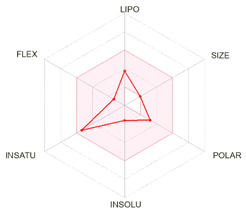	Yes; 0 violation	No; 1 violation: MW < 250	2.48
20	Caffeic acid	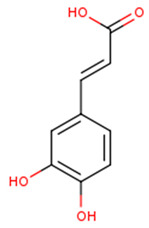	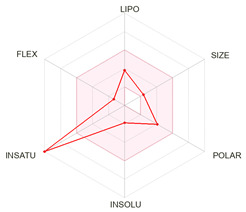	Yes; 0 violation	No; 1 violation: MW < 250	1.81
21	Acteoside	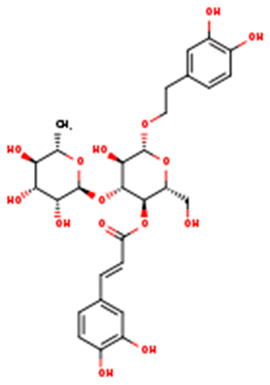	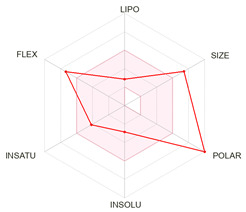	No; 3 violations: MW > 500, NorO > 10, NHorOH > 5	No; 2 violations: MW > 350, Rotors > 7	6.37
22	6-hydroxysalvinolone	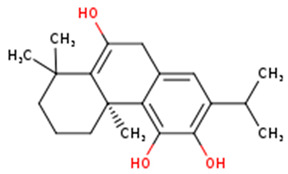	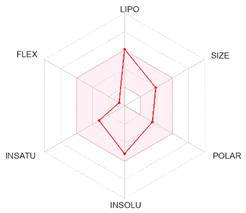	Yes; 0 violation	No; 1 violation: XLOGP3 > 3.5	3.94
23	Citronellol	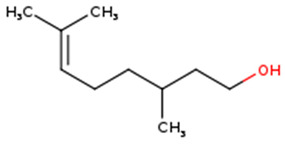	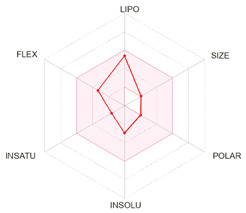	Yes; 0 violation	No; 2 violations: MW < 250, XLOGP3 > 3.5	2.61
24	Clerodin	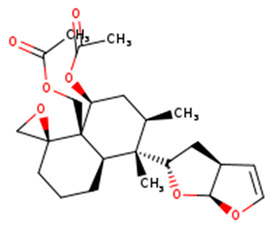	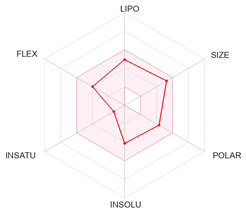	Yes; 0 violation	No; 1 violation: MW > 350	6.39
25	Clerodendrin A	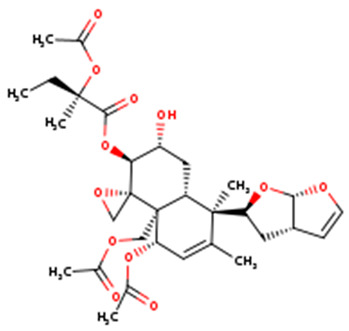	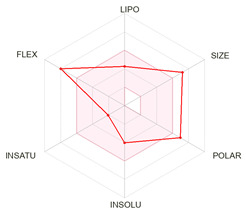	No; 2 violations: MW > 500, NorO > 10	No; 2 violations: MW > 350, Rotors > 7	7.47
26	Scroside E	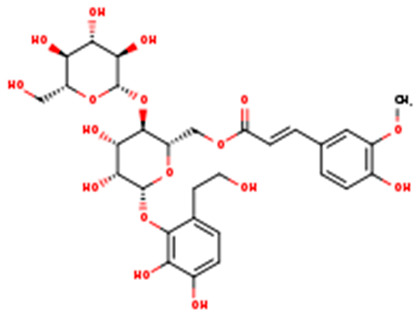	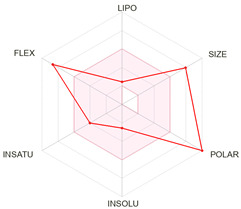	No; 3 violations: MW > 500, NorO > 10, NHorOH > 5	No; 2 violations: MW > 350, Rotors > 7	6.72
27	Campneoside I	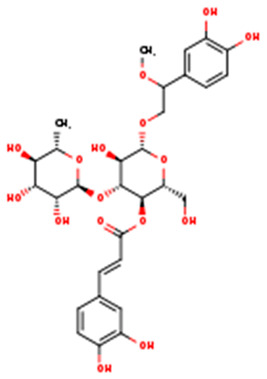	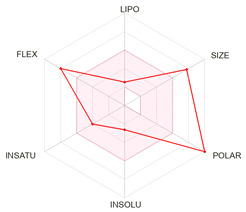	No; 3 violations: MW > 500, NorO > 10, NHorOH > 5	No; 2 violations: MW > 350, Rotors > 7	6.60
28	Forsythoside B	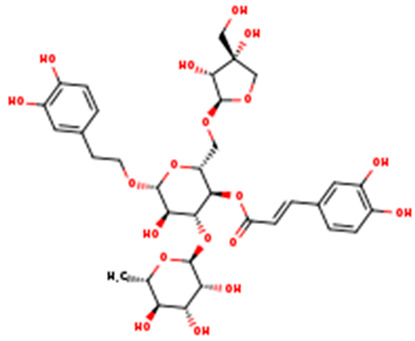	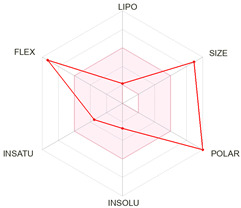	No; 3 violations: MW > 500, NorO > 10, NHorOH > 5	No; 2 violations: MW > 350, Rotors > 7	7.23
29	Forsythoside A	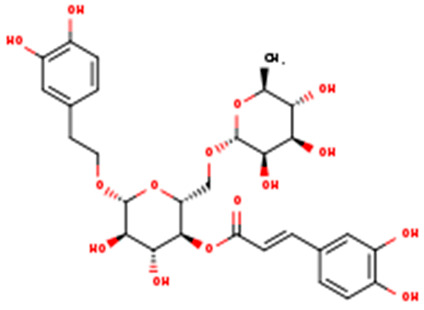	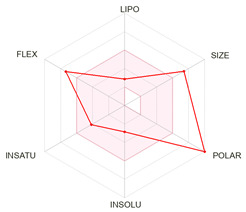	No; 3 violations: MW > 500, NorO > 10, NHorOH > 5	No; 2 violations: MW > 350, Rotors > 7	6.37
30	Isoacteoside	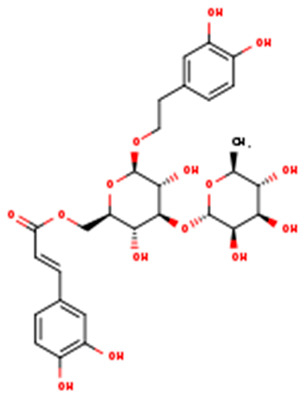	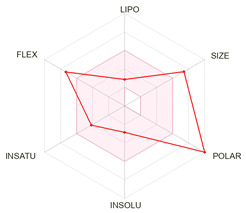	No; 3 violations: MW > 500, NorO > 10, NHorOH > 5	No; 2 violations: MW > 350, Rotors > 7	6.37
31	Lavandulifolioside	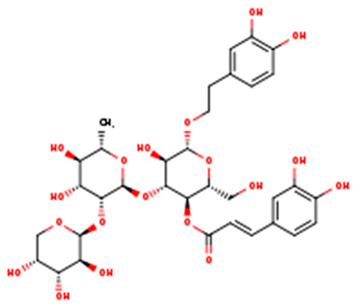	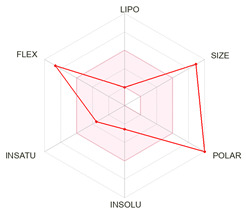	No; 3 violations: MW > 500, NorO > 10, NHorOH > 5	No; 2 violations: MW > 350, Rotors > 7	7.28
32	Nobilin D	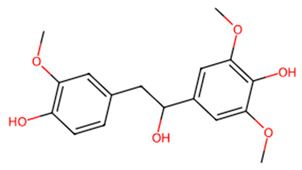	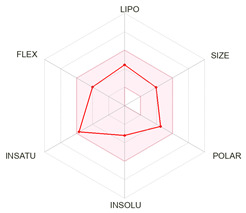	Yes; 0 violation	Yes	2.94
33	4-Hydroxy-3-methoxycinnamic acid	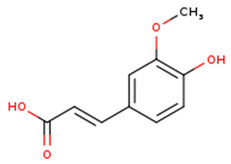	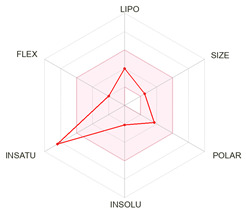	Yes; 0 violation	No; 1 violation: MW < 250	1.93
34	Linarin	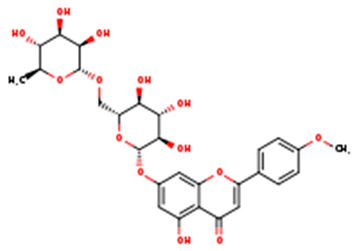	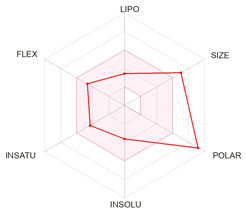	No; 3 violations: MW > 500, NorO > 10, NHorOH > 5	No; 1 violation: MW > 350	6.43
35	Peonidin-3,5-O-di-beta-glucopyranoside	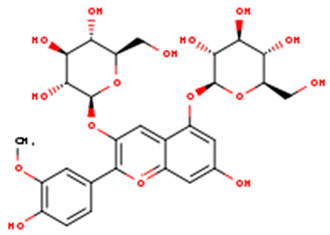	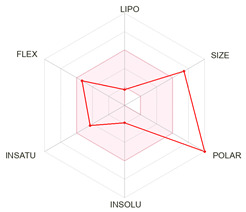	No; 3 violations: MW > 500, NorO > 10, NHorOH > 5	No; 2 violations: MW > 350, Rotors > 7	6.67
36	trans-Cinnamic acid	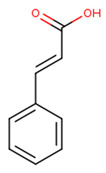	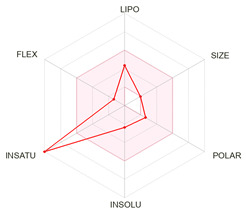	Yes; 0 violation	No; 1 violation: MW < 250	1.67
37	Daidzein	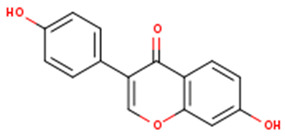	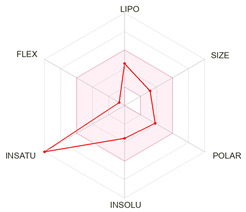	Yes; 0 violation	Yes	2.79
38	Saponarin	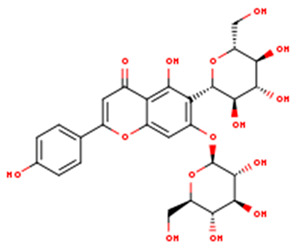	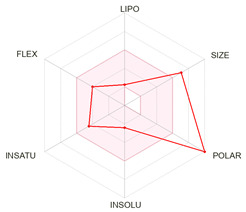	No; 3 violations: MW > 500, NorO > 10, NHorOH > 5	No; 1 violation: MW > 350	6.38
39	Homoorietin	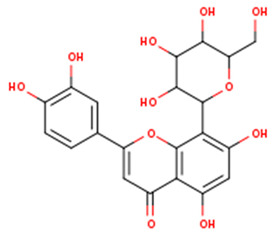	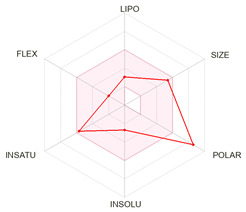	No; 2 violations: NorO > 10, NHorOH > 5	No; 1 violation: MW > 350	5.17
40	Acacetin	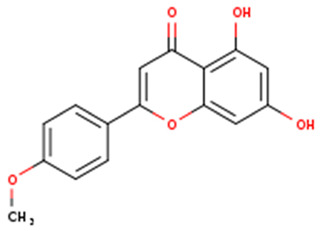	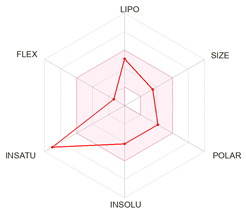	Yes; 0 violation	Yes	2.98
41	Sarsasapogenin	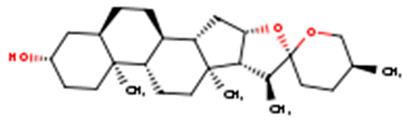	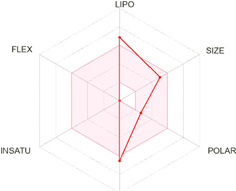	Yes; 1 violation: MLOGP > 4.15	No; 2 violations: MW > 350, XLOGP3 > 3.5	6.88
42	Phytol	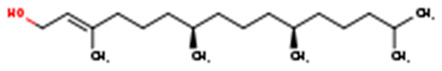	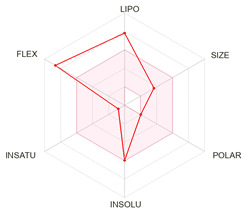	Yes; 1 violation: MLOGP > 4.15	No; 2 violations: Rotors > 7, XLOGP3 > 3.5	4.30
43	Sissotrin	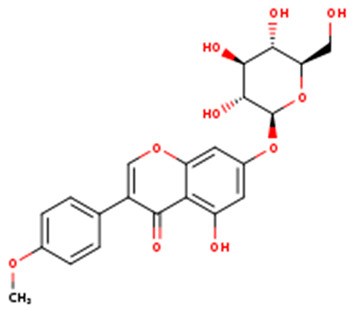	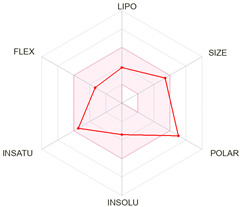	Yes; 0 violation	No; 1 violation: MW > 350	5.23

**Table 6:** Pharmacological Evidence, Target Validation Status, and Experimental Support for Selected Bioactive Compounds.

No	Compound Name	Compound Source	Evidence Type	Target Status	Validation Method	Test Material	Reference
1	Luteolin	Key bioactive compound of Sanhua Decoction identified through TCMSP-based network pharmacology	Direct compound-level evidence	Predicted through network pharmacology and experimentally validated through in vitro assays.	TCMSP screening, OMIM and DisGeNET disease-target collection, Venny 2.1 intersection analysis, STRING PPI network, Metascape enrichment analysis, Cytoscape software (Version 3.9.1) network construction, CCK-8 assay, scratch assay, Transwell migration assay, invasion assay, tube formation assay, and Western blot analysis.	Pure compound luteolin; VEGF165-induced HUVECs	[86]
2	Acacetin	Naturally occurring O-methylated flavone reported from several edible and medicinal plants, including *Robinia pseudoacacia*, bee propolis, *Dracocephalum moldavica*, *Turnera diffusa*, and *Betula pendula*	Direct and indirect compound-level evidence	EGFR, STAT3, AKT, PI3K, and PTGS2 were supported by docking and experimental validation, while SRC, HSP90AA1, TNF, IL-6, and MYC were mainly supported by docking-only evidence.	Network pharmacology, molecular docking, DARTS, CETSA, pull-down assay, kinase assay, Western blot, CCK-8, Annexin V/PI, TUNEL, colony formation, migration and invasion assays, and xenograft models.	Pure compound acacetin; multiple cancer cell lines and xenograft models	[87]
3	Tricin	Main bioactive compound of Weijing decoction, mainly associated with *Rhizoma Phragmitis* and confirmed in the formula by UPLC-Q-TOF/MS	Direct compound-level evidence	PRKCA was predicted by network pharmacology and experimentally validated; SPHK1/SPHK2/S1P signaling was supported by metabolomics and molecular assays.	TCMSP, BATMAN-TCM, CTD, DisGeNET, Cytoscape network construction, pathway enrichment analysis, UPLC-Q-TOF/MS, plasma metabolomics, MTT assay, colony formation assay, wound healing assay, apoptosis assay, qRT-PCR, Western blot, sh-Prkca knockdown, xenograft assay, survival assay, IHC, and H&E staining.	Pure compound Tricin; Weijing decoction; LLC cells, human NSCLC cell lines, CCD-19Lu normal lung fibroblasts, and LLC xenograft mouse model	[88]
4	Kaempferide	Natural antioxidant screened from natural products using TCMSP and SymMap based on the FoxO1/β-catenin signaling pathway	Direct compound-level evidence	GSK-3β and JNK1 were predicted as kaempferide targets by systems pharmacology and supported by molecular docking/MD simulation; FoxO1/β-catenin signaling was experimentally supported in vitro.	Systems pharmacology, reverse drug targeting, ADME screening, target-compound-function network analysis, molecular docking, MD simulation, MTT assay, ALP staining, alizarin red staining, qRT-PCR, Western blot, and H_2_O_2_-induced oxidative injury model.	Pure compound kaempferide; MC3T3-E1 preosteoblast cells	[89]
5	Glycitein	Soy-derived O-methylated isoflavone identified as a candidate compound through CMap screening of TCGA-COAD differentially expressed genes	Computational compound-level evidence	CCNA2, ESR1, ESR2, MAPK14, and PTGS2 were predicted by network pharmacology and supported by molecular docking; PTGS2 showed the strongest docking affinity.	TCGA-COAD DEG analysis, CMap screening, TCMSP target retrieval, MalaCards disease-gene collection, STRING PPI network, random walk with restart analysis, Cytoscape network construction, GO/KEGG enrichment, and SwissDock molecular docking.	In silico colon cancer model based on TCGA-COAD dataset; glycitein as pure compound structure from PubChem	[90]
6	Daidzein	Soy-derived phytoestrogenic isoflavone also reported from soybean, red clover, and alfalfa	Direct and computational compound-level evidence	EGFR, CDK6, GSK3B, FLT3, HSP90AA1, HSP90AB1, NTRK1, MCL1, and RAF1 were predicted as GBM-related targets and supported by molecular docking; PI3K-Akt was identified as the major enriched pathway.	SwissADME, SwissTargetPrediction, SuperPred, OMIM, GeneCards, Venny, STRING, Cytoscape, MCODE, GO/KEGG enrichment, GEPIA2 survival analysis, molecular docking using Schrödinger Maestro, antioxidant assays, and MTT assay.	Pure compound daidzein; chicken brain tissue homogenate; SH-SY5Y human neuroblastoma cells	[91]
7	Nobilin D	A major compound reported from *Dendrobium nobile* and identified as a key pharmacodynamic material based on the highest degree value in the compound-target network	Computational compound-level evidence	MIF, ESR2, CYP19A1, ABCG2, TLR9, and DRD5 were predicted as key targets; ESR2, MIF, ABCG2, and CYP19A1 were further supported by molecular docking.	Literature-based compound collection, TargetNet prediction, CTD disease association, DAVID GO/KEGG enrichment, Cytoscape network analysis, STRING PPI network, and AutoDock Vina molecular docking	In silico model of *Dendrobium nobile* compounds and nervous system disease-related targets	[92]
8	Pectolinarigenin	Natural metabolite reported from *Cirsium setidens* Nakai and selected from an antioxidant compound library	Direct compound-level evidence	HIF-1α was predicted by network pharmacology and experimentally validated as a direct binding target; pectolinarigenin inhibited HIF-1α-mediated inflammation and oxidative stress.	Antioxidant compound screening, CCK-8 assay, qRT-PCR, Western blot, immunofluorescence, GeneCards-DAVID network pharmacology, GO/KEGG enrichment, molecular docking, luciferase reporter assay, CETSA, HIF-1α knockdown, ROS/GSH/MDA/iron assays, and CaOx nephrocalcinosis mouse model	Pure compound pectolinarigenin; COM-stimulated HK-2 cells; glyoxylate-induced CaOx nephrocalcinosis mouse model.	[93]
9	Syringetin	Flavonoid derivative evaluated as a candidate compound for breast cancer-related bone pain	Direct compound-level evidence	ESR1 was identified as the core target by network pharmacology and MCODE analysis, supported by molecular docking, and functionally linked with PRDM2 suppression and reduced neuronal activation.	SEA, PharmMapper, HERB, TCMSP, SwissTargetPrediction, GeneCards, NCBI, OMIM, Venny, STRING, Cytoscape, MCODE, KEGG enrichment, molecular docking using AutoDock Vina, CCK-8 assay, TUNEL assay, Transwell migration assay, Western blot, HE staining, pain behavioral tests, and immunohistofluorescence.	Pure compound syringetin; SHZ-88 rat breast cancer cells; breast cancer-related bone pain rat model induced by SHZ-88 cell implantation.	[94]

**Table 7:** Network Pharmacology Strategies, Target Identification, and Pharmacological Activities of Bioactive Compounds.

**No**	**Compound Name**	**Methodological Approach**	**Disease Type**	**Target Database**	**Selected Target**	**Research Finding**	**Reference**
1	Luteolin	The study employed a combination of network pharmacology and in vitro experimental validation. Analytical methods included PPI network construction, enrichment analysis, and biological assays such as CCK-8, migration, invasion, tube formation, and Western blot.	The study focused on Macular Degeneration. This condition is characterized by abnormal angiogenesis associated with VEGFA overexpression.	Active compounds were obtained from TCMSP, while disease-related targets were collected from OMIM and DisGeNET. Additional analyses utilized UniProt, STRING, Metascape, and Venny 2.1.	Six key targets were identified, including ESR1, PON1, SOD1, APOB, VEGFA, and IL6. Among these, VEGFA was selected as the primary target due to its central role in angiogenesis.	Luteolin showed an IC_50_ value of 101.4 μM and an IC_10_ value of 25.26 μM in HUVECs, with 25 μM used as the working concentration. It significantly inhibited cell migration, invasion, and tube formation, while also reducing VEGFA expression, indicating strong anti-angiogenic activity.	[86]
2	Acacetin	The study employed an integrated approach combining network pharmacology, molecular docking, and experimental validation (in vitro and in vivo). Computational predictions were supported by techniques such as PPI analysis, docking simulations, Western blot, xenograft models, and functional assays.	The study focused on Cancer Therapy. It covered multiple cancer types, including breast, lung, colorectal, gastric, liver, and prostate cancers.	Target prediction and validation involved databases such as SwissTargetPrediction, TCMSP, GeneCards, OMIM, DisGeNET, and STRING, with network visualization using Cytoscape. Docking analyses were conducted using tools such as AutoDock Vina and Schrödinger Maestro.	Key targets identified and validated include EGFR, STAT3, and AKT1, which act as central hub proteins in oncogenic signaling pathways. Additional targets such as PI3K, PTGS2, SRC, HSP90AA1, TNF, IL-6, and MYC were also predicted or partially validated.	Docking results showed strong binding affinities, including AKT1 (−9.2 kcal/mol), STAT3 (≈−9.0 kcal/mol), and EGFR (−8.3 kcal/mol), indicating stable interactions. Acacetin demonstrated significant anticancer effects by inducing apoptosis, inhibiting proliferation, suppressing metastasis, and reducing angiogenesis through modulation of EGFR/STAT3/AKT signaling pathways.	[87]
3	Tricin	The study utilized an integrated approach combining network pharmacology, metabolomics (UPLC-Q-TOF/MS), and biological validation (in vitro and in vivo experiments). Additional analyses included PPI network construction, KEGG pathway enrichment, xenograft mouse models, and molecular assays such as Western blot and qPCR.	The research focused on Non-Small Cell Lung Cancer (NSCLC), which represents the most prevalent form of lung cancer with high mortality rates.	Target identification and analysis involved databases such as TCMSP, BATMAN-TCM, CTD, DisGeNET, and STRING, with visualization and network construction performed using Cytoscape.	Key targets include PRKCA (Protein Kinase C alpha) as the central regulator, along with related proteins such as PRKCB, SPHK1, and SPHK2, which are involved in sphingolipid signaling pathways.	The study demonstrated that Tricin significantly inhibits tumor growth by suppressing PRKCA/SPHK/S1P signaling pathways, reducing cell proliferation, migration, and colony formation while inducing apoptosis. In vivo experiments showed strong tumor inhibition, with high-dose Tricin achieving up to 65.03% tumor inhibition, indicating its potential as an alternative anticancer agent.	[88]
4	Kaempferide	The study used an integrated systems pharmacology and in vitro validation approach. The workflow included reverse drug targeting, ADME screening, network analysis, molecular docking, molecular dynamics simulation, and biological assays such as ALP staining, alizarin red staining, qPCR, and Western blot.	The study focused on Osteoporosis Treatment. This disease is closely associated with oxidative stress and impaired osteogenic differentiation	Target and compound screening were performed using TCMSP and SymMap, while structural information was obtained from PubChem and RCSB PDB. Network analysis was conducted with Cytoscape.	The major selected targets were JNK1 and GSK-3β, which regulate the FoxO1/β-catenin signaling pathway. Downstream antioxidant-related targets included SOD2, Catalase, and GPx-1.	Kaempferide showed favorable ADME properties with OB = 73.41%, DL = 0.27, and Caco-2 = 0.43, and it was identified as the most potent OP-related antioxidant. In vitro, 10 μM kaempferide significantly enhanced ALP activity, mineralization, antioxidant gene expression, and nuclear translocation of FoxO1 and β-catenin, while also rescuing H_2_O_2_-induced impairment of osteogenesis.	[89]
5	Glycitein	The study applied network pharmacology combined with molecular docking. Key methods included DEG analysis from TCGA, CMap screening, PPI network construction, RWR analysis, GO/KEGG enrichment, and docking validation using SwissDock.	The study focused on Colon Cancer. This disease is characterized by malignant transformation of colonic epithelial cells and high global incidence and mortality	Targets and genes were obtained from TCGA (TCGA-COAD), CMap, TCMSP, MalaCards, STRING, and PubChem, with enrichment analysis using R packages such as clusterProfiler.	Five core targets were identified, including CCNA2, ESR1, ESR2, MAPK14, and PTGS2. These targets were further analyzed as seed genes in RWR-based network analysis.	Molecular docking showed spontaneous binding of glycitein to all targets, with the strongest interaction observed for PTGS2 (ΔG = −8.62 kcal/mol), followed by ESR2 (−7.92 kcal/mol). Enrichment analysis revealed involvement in key pathways such as MAPK, PI3K-Akt, FoxO, and p53 signaling, indicating glycitein’s role in regulating proliferation, apoptosis, and tumor progression.	[90]
6	Daidzein	The study used an integrated approach combining network pharmacology, molecular docking, and in vitro validation. Methods included target prediction (SwissTargetPrediction, SuperPred), PPI analysis (STRING, Cytoscape), GO/KEGG enrichment, docking using Maestro (Schrödinger), antioxidant assays (SOD, GSH, TBARs), and MTT cytotoxicity testing.	The study focused on Glioblastoma (GBM), a highly aggressive grade IV brain tumor with poor prognosis and high resistance to conventional therapies.	Targets were obtained from PubChem, SwissTargetPrediction, SuperPred, OMIM, GeneCards, STRING, GEPIA2, and enrichment tools. These databases enabled the identification of overlapping DDZ–GBM targets and pathway analysis.	A total of 21 intersecting targets were identified, with key hub genes including EGFR, CDK6, GSK3B, FLT3, NTRK1, HSP90AA1, HSP90AB1, MCL1, RAF1, ESR1, and PPARG. These were central in the PPI network and PI3K-Akt signaling.	KEGG analysis revealed that the PI3K-Akt signaling pathway was the most significantly enriched (adjusted *p* = 2.68 × 10^−6^), involving multiple oncogenic targets linked to proliferation, survival, and apoptosis resistance. Molecular docking showed strong binding affinities (−7.2 to −9.1 kcal/mol) with key proteins such as EGFR, CDK6, GSK3B, and HSP90 isoforms, indicating multitarget anticancer potential. In vitro results demonstrated significant antioxidant effects (increase in SOD and GSH, decrease in TBARs) and low cytotoxicity at moderate doses (>94% viability at 200 µg/mL), supporting both neuroprotective and anticancer roles of daidzein.	[91]
7	Nobilin D	The study employed network pharmacology combined with molecular docking. Methods included target prediction (TargetNet), network construction (Cytoscape), PPI analysis (STRING), enrichment analysis (DAVID), and docking validation using AutoDock Vina.	The study focused on Nervous System Diseases. These include neurodegenerative and neuroinflammatory disorders such as Alzheimer’s disease, Parkinson’s disease, and epilepsy.	Targets and pathways were obtained from TCMSP, PubChem, TargetNet, SwissTargetPrediction, UniProt, CTD, STRING, DAVID, and OMIM. These databases enabled comprehensive compound–target–disease network analysis.	Key targets identified include MIF, ESR2, CYP19A1, ABCG2, TLR9, and DRD5, along with hub proteins such as APP, SRC, CASP3, TNF, and PTGS2 from PPI analysis. These targets are involved in inflammation, neurotransmission, and neurodegeneration.	Network analysis revealed that Dendrobium nobile acts through multi-component and multi-target mechanisms, with key pathways including sphingolipid signaling, serotonergic synapse, and cocaine addiction pathways. Molecular docking showed strong binding affinities (generally < −5 kcal/mol) with stable interactions between key compounds and targets such as ESR2 and MIF.	[92]
8	Pectolinarigenin	The study used an integrated approach combining network pharmacology, molecular docking, CETSA, and in vitro/in vivo validation. Experimental validation included HK-2 cell assays, a glyoxylate-induced mouse nephrocalcinosis model, PCR, Western blot, immunofluorescence, luciferase reporter assay, and oxidative stress measurements.	The study focused on CaOx Nephrocalcinosis. This condition is characterized by calcium oxalate crystal deposition, renal tubular injury, inflammation, and oxidative stress.	Targets and pathways were analyzed using GeneCards and DAVID, with molecular interaction further confirmed by Discovery Studio docking. These tools were used to identify key pathways and candidate molecular targets of pectolinarigenin.	The main selected target was HIF-1α, identified through network pharmacology and KEGG enrichment analysis. Additional pathway-related genes analyzed included EGFR, AKT, IGF-1R, and NOS2.	Pectolinarigenin significantly reduced renal crystal deposition, tubular injury, inflammatory cytokine expression, ROS, MDA, and iron levels, while increasing GSH, HO-1, and GPX4 in both cells and mice. Molecular docking, CETSA, and luciferase reporter assays showed that pectolinarigenin directly binds to HIF-1α and inhibits its activity, thereby alleviating calcium oxalate-induced renal inflammation and oxidative stress.	[93]
9	Syringetin	The study applied an integrated approach combining network pharmacology, molecular docking, in vitro cell experiments, and in vivo animal models. Validation methods included CCK-8 assay, TUNEL assay, Transwell migration assay, Western blotting, immunofluorescence, HE staining, behavioral pain tests (AS and PWT), and bioinformatics analysis.	The study focused on breast cancer-related bone cancer pain (BCP), a complication of metastatic breast cancer characterized by bone destruction, inflammation, and neural sensitization.	Target identification and analysis were performed using PubChem, SEA, PharmMapper, HERB, TCMSP, SwissTargetPrediction, GeneCards, NCBI, OMIM, STRING, and Cytoscape, with KEGG pathway enrichment analysis for biological interpretation.	The core target identified was ESR1 (estrogen receptor 1). Other relevant targets included TERT, AKT1, EGFR, BST1, ALDH2, and PLA2G2A, with ESR1 emerging as the key hub gene through MCODE and PPI analysis.	Syringetin showed strong binding affinity to ESR1 (−7.5 kcal/mol) and formed hydrogen bonds with key residues such as ASN222, GLY220, and THR470. It significantly inhibited breast cancer cell viability, migration, and ESR1/PRDM2 expression while promoting apoptosis. In vivo, syringetin reduced tumor burden, preserved bone structure, improved pain behavior (AS and PWT), and decreased ESR1 and PRDM2 expression in bone tissue. Additionally, it suppressed spinal cord neuronal activation (GFAP, IBA1, NeuN), with ESR1 positively correlated with these neural markers, indicating that syringetin alleviates bone cancer pain via the ESR1/PRDM2 axis and modulation of neural signaling pathways.	[94]

## Data Availability

The original contributions presented in this study are included in the article. Further inquiries can be directed to the corresponding author.

## Abbreviations

The following abbreviations are used in this manuscript:

AAE	Ascorbic Acid Equivalent
ACAE	Acarbose Equivalent
AgNPs	Silver Nanoparticles
BSL	Brine Shrimp Lethality
COX	Cyclooxygenase
DPPH	2,2-Diphenyl-1-picrylhydrazyl
FRAP	Ferric Reducing Antioxidant Power
FTIR	Fourier Transform Infrared Spectroscopy
GAE	Gallic Acid Equivalent
GC-MS	Gas Chromatography–Mass Spectrometry
HPLC	High Performance Liquid Chromatography
HRMS	High-Resolution Mass Spectrometry
IC_50_	Half Maximal Inhibitory Concentration
Ki	Inhibition Constant
LC-MS	Liquid Chromatography–Mass Spectrometry
LC_50_	Lethal Concentration 50%
MIC	Minimum Inhibitory Concentration
MTT	3-(4,5-Dimethylthiazol-2-yl)-2,5-diphenyltetrazolium bromide
NMR	Nuclear Magnetic Resonance
PDA	Potato Dextrose Agar
QE	Quercetin Equivalent
ROS	Reactive Oxygen Species
SEM	Scanning Electron Microscopy
SPF	Sun Protection Factor
TFC	Total Flavonoid Content
TLC	Thin Layer Chromatography
TPC	Total Phenolic Content
UHPLC	Ultra-High Performance Liquid Chromatography
UPLC	Ultra Performance Liquid Chromatography
UV–Vis	Ultraviolet–Visible Spectroscopy
XRD	X-ray Diffraction
